# ILDR2: An Endoplasmic Reticulum Resident Molecule Mediating Hepatic Lipid Homeostasis

**DOI:** 10.1371/journal.pone.0067234

**Published:** 2013-06-24

**Authors:** Kazuhisa Watanabe, Elizabeth Watson, Maria Laura Cremona, Elizabeth J. Millings, Jay H. Lefkowitch, Stuart G. Fischer, Charles A. LeDuc, Rudolph L. Leibel

**Affiliations:** 1 Naomi Berrie Diabetes Center and Department of Pediatrics, Columbia University, New York, New York, United States of America; 2 Department of Pathology and Cell Biology, Columbia University, New York, New York, United States of America; University of Salento, Italy

## Abstract

*Ildr2,* a modifier of diabetes susceptibility in obese mice, is expressed in most organs, including islets and hypothalamus, with reduced levels in livers of diabetes-susceptible B6.DBA mice congenic for a 1.8 Mb interval of Chromosome 1. In hepatoma and neuronal cells, ILDR2 is primarily located in the endoplasmic reticulum membrane. We used adenovirus vectors that express shRNA or are driven by the CMV promoter, respectively, to knockdown or overexpress *Ildr2* in livers of wild type and *ob/ob* mice. Livers in knockdown mice were steatotic, with increased hepatic and circulating triglycerides and total cholesterol. Increased circulating VLDL, without reduction in triglyceride clearance suggests an effect of reduced hepatic ILDR2 on hepatic cholesterol clearance. In animals that overexpress *Ildr2*, hepatic triglyceride and total cholesterol levels were reduced, and strikingly so in *ob/ob* mice. There were no significant changes in body weight, energy expenditure or glucose/insulin homeostasis in knockdown or overexpressing mice. Knockdown mice showed reduced expression of genes mediating synthesis and oxidation of hepatic lipids, suggesting secondary suppression in response to increased hepatic lipid content. In *Ildr2*-overexpressing *ob/ob* mice, in association with reduced liver fat content, levels of transcripts related to neutral lipid synthesis and cholesterol were increased, suggesting “relief” of the secondary suppression imposed by lipid accumulation. Considering the fixed location of ILDR2 in the endoplasmic reticulum, we investigated the possible participation of ILDR2 in ER stress responses. In general, *Ildr2* overexpression was associated with increases, and knockdown with decreases in levels of expression of molecular components of canonical ER stress pathways. We conclude that manipulation of *Ildr2* expression in liver affects both lipid homeostasis and ER stress pathways. Given these reciprocal interactions, and the relatively extended time-course over which these studies were conducted, we cannot assign causal primacy to either the effects on hepatic lipid homeostasis or ER stress responses.

## Introduction

In an earlier study [1] we exploited the differential diabetes susceptibilities of mouse strains C57BL/6J (B6) and DBA/2J (DBA) [2] segregating for the obesity mutation, *Lep^ob^*, to identify a gene that encodes a predicted single-pass, trans-membrane molecule that, in B6.DBA congenic mice (segregating a DBA haplotype in a 1.8 Mb interval on Chr1), was associated with reduced β-cell replication rates accompanied by reduced β-cell mass, and persistent mild hypoinsulinemic hyperglycemia. This gene, formerly designated “*Lisch-like”,* has been renamed “immunoglobulin-like domain containing receptor 2″ (*Ildr2*) [http://www.informatics.jax.org/mgihome/nomen/] to reflect the similarity of the conserved domain structure of the cognate protein to the two other members of this gene family: *Ildr1* and *Ildr3* (aka “LSR” – lipolysis stimulated receptor).

Despite their structural similarities, the three *Ildr*-genes exhibit widely divergent tissue-specific expression profiles, providing little evidence of significant overlap among their functions. The major isoforms of both ILDR1 and ILDR3 localize either to the plasma membrane (PM) or to the cytosol [3], [4]. Although ILDR1 has been linked to neoplastic disease 2 [5] and non-syndromic deafness [6], how it functions is unknown. ILDR3, which was initially identified as a fatty acid-activated, liver-specific lipoprotein receptor [7], has since been characterized variously as a receptor for *Clostridium* toxin [8], as an hepatic receptor upregulated by leptin [9] and as a component of tri-cellular junctions in epithelial cells [10].

The *Ildr2* gene is widely expressed, with 4 major isoforms that are differentially expressed in tissues relevant to the diabetic phenotype (hypothalamus, liver and islet β-cells). Expression levels of isoform 4, highest in liver, are reduced 20-fold in B6.DBA congenic animals and 30-fold in 10-week-old DBA mice versus B6 animals [1]. To assess the role of *Ildr2* in the molecular physiology of normal, adult liver, we used intravenously administered adenoviruses containing overexpression or knockdown constructs to study *in vivo* effects in liver and whole animal, and in transduced primary hepatocytes to study *in vitro* effects.

Here we report that ILDR2, in contrast to ILDR1 and ILDR3, is exclusively localized in the endoplasmic reticulum (ER), where it apparently participates in both lipoprotein physiology and the ER stress response, with consequences for hepatic lipid homeostasis.

## Results

### ILDR2 is Localized to the Endoplasmic Reticulum

**Figure 1:**
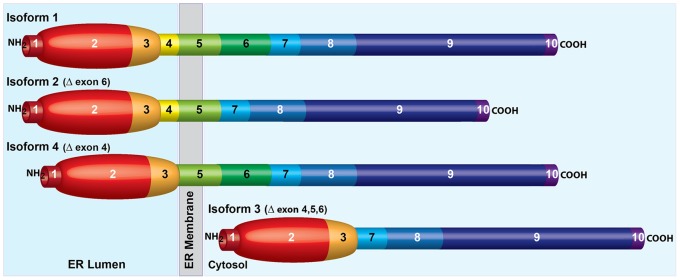
Predicted structure of major ILDR2 isoforms. Isoform 1 (GenBank: FJ024495.1) is full-length. There are 10 predicted exons. Exon 1 is an amino terminal signal peptide; exons 2 and 3 code for an IgV-like immunoglobulin domain; exon 4 is amino proximal to the trans-membrane domain of exon 5; exons 6–10 comprise a randomly-coiled, carboxy-terminal tail (simplified in this depiction as rod-like). Based on results shown in **Figure 2**, exons 1–4 are lumenal and exons 6–10 are cytosolic. Isoform 2 (GenBank: FJ024496.1) lacks cytosolic exon 6. Isoform 4 (GenBank: FJ024498.1) lacks lumenal exon 4. Isoform 3 (GenBank: FJ024497.1) lacks exons 4, 5, and 6 and, therefore has no trans-membrane domain, and is depicted as entirely cytosolic.

As previously described [1], the four major isoforms of ILDR2 (**Figure 1**) contain an amino terminal immunoglobulin-like domain and long, carboxy tail. Isoforms 1, 2, and 4 also contain a single trans-membrane (TM) domain. Isoform 1 is full-length; isoform 2 lacks exon 6 (carboxy to the TM domain); isoform 4 lacks exon 4 (amino to the TM domain); isoform 3 lacks the TM domain and both flanking exons.

**Figure 2:**
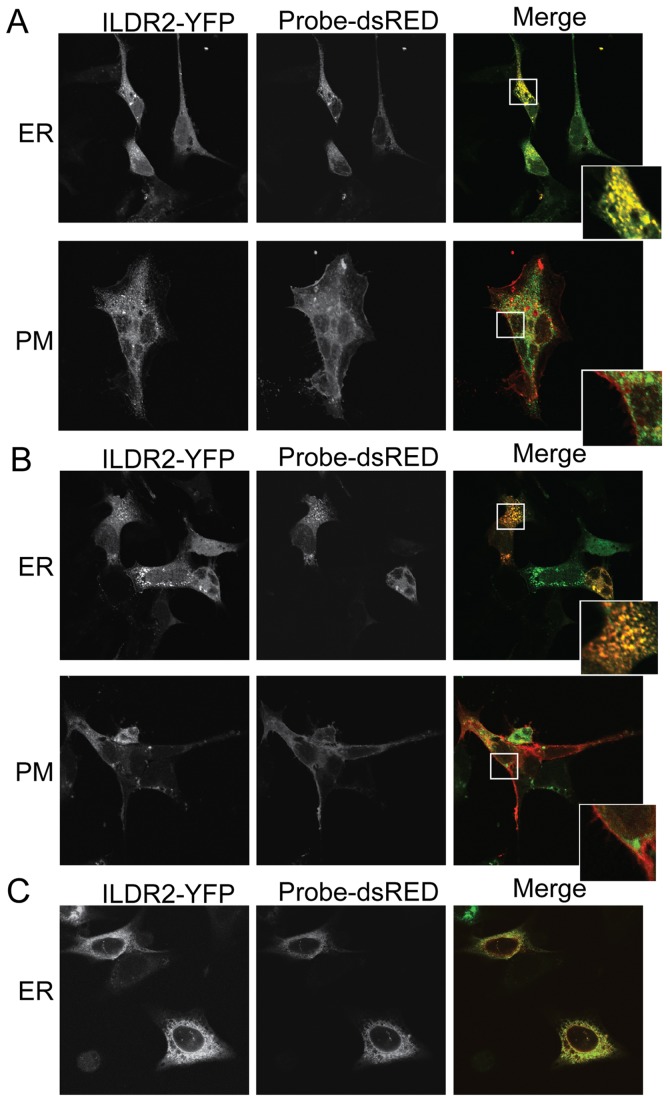
Fluorescence microscopy of ILDR2 localization under basal conditions. ILDR2 fused on its C-terminus to mYFP (green) was transiently co-transduced into cell lines with DsRed-probes specific to either the ER (red) or the PM (red). The ER-specific probe is DsRed fluorescent protein attached to the ER-retention sequence KDEL. The PM-specific probe is DsRED attached to a farnesyl group that targets the protein to the inner leaflet of the PM. Cells were fixed without any further treatment 24 hr after transfection. Bar: 100 uM. Confocal images recorded at 63× magnification. (A) GT1-7 cells. ILDR2-isoform 2-YFP merges with DsRed-ER probe to produce a yellow signal over the ER, but does not merge with the red DsRed-PM probe. (B) Hepa1c1c7 cells. The green ILDR2-isoform 4-YFP probe merges with the red DsRed-ER probe to produce an orange signal over the ER; expression levels of labeled proteins are less uniform than in GT1-7 cells. The red DsRed-PM and green ILDR2-YFP signals do not merge in the PM. (C) Hepa1c1c7 cells. N-terminal fusion of ILDR2-isoform 1 with 3xFLAG epitope co-transduced with DsRed-probes to ER. Tag geometry does not interfere with subcellular localization.

To determine the cellular location(s) of ILDR2, various isoforms were tagged at the C-termini with the green variant of the monomeric yellow fluorescent protein (mYFP), transiently transduced into mouse cells, and analyzed by confocal microscopy for co-localization with probes for the ER and PM **(** **Figure 2** **)**. *Ildr2*-isoform 2, predominantly expressed in the hypothalamus, was transduced into cells of the mouse hypothalamic neuronal cell line GT1-7 **(** **Figure 2A** **)**. *Ildr2*-isoform 4, the predominant isoform endogenously expressed in the liver, was transduced into cells of the mouse hepatoma cell line, Hepa1c1c7 **(** **Figure 2B** **)**. Both isoforms localized solely to the ER membrane, with no detectable fluorescence in the vicinity of the PM. Placement of the tagging peptide did not affect subcellular destination, since localization to the ER membrane was seen also in Hepa1c1c7 cells transduced with *Ildr2*-isoform 1 tagged at its N-terminus with the FLAG epitope **(** **Figure 2C** **)**. These results support the model depicted in **Figure 1** **,** in which the hydrophobic, amino-terminal, immunoglobulin-like domain of isoforms 1, 2 and 4, extends into the ER lumen, and the hydrophilic carboxy-terminal tail, extends into the cytoplasm. We also observed no changes in the subcellular distribution of C-terminal tagged isoform 4 in Hepa1c1c7 transfectants that were exposed to glucose, insulin, free fatty acids (FFA), and low-density lipoprotein (LDL) (data not shown). These results suggest that, unlike ILDR1 and ILDR3, whose final destination is the PM, ILDR2 is an integral ER trans-membrane molecule that likely does not further translocate within these cell types.

### Functional Analysis of Ildr2

The ER plays critical roles in protein and lipid synthesis, lipoprotein assembly and export, glucose and calcium homeostasis, and cellular responses to metabolic stress [11]–[15]. These protean functions affect liver [16], hypothalamus [17], and β-cells [18]. The metabolic phenotypes seen in the *Ildr2* B6.DBA.congenic lines are consistent with effects on ER stress mechanisms [1]. Accessibility of the liver to *in vivo* and *in vitro* transcriptional manipulation using adenovirus vectors [19], [20], led us to focus on the liver.

To examine the effects of short term changes in *Ildr2* expression in liver on lipid and glucose homeostasis, 10-week-old chow-fed C57BL/6J (wild-type; WT) or B6.Cg-Lep*^ob^*/J (obese; OB) male mice were transduced with adenoviral expression vectors encoding shRNA (“ADKD”) that knockdown *Ildr2*, or with adenoviral constructs driven by the CMV promoter (“ADOX”) that overexpress *Ildr2*. To control for non-specific effects of adenoviral transduction on gene expression, we also transduced mice with adenoviral expression vectors encoding shRNA that knockdown *lacZ*, or with adenoviral constructs driven by the CMV promoter that overexpress the green fluorescent protein (*GFP*). Expression levels in the hypothalamus and white adipose tissue were unaffected by transduction with either the ADKD or ADOX viral constructs (data not shown), confirming that their effects were restricted primarily to the liver. Knockdown efficiency exceeded 80% at 3 days post-transduction (p.t.) and 90% at 10 days p.t., while *Ildr*2 overexpression resulted in 2- to 4-fold increases in mRNA levels.

For studies of liver morphology, histology and chemistry, and for liver-specific gene expression analysis, animals were sacrificed at 3 days or 10 days p.t. To provide a general picture of the cellular/biochemical consequences of manipulations of expression of hepatic *Ildr2*, we evaluated the livers by visual inspection, light microscopy, chemical composition, and by quantitative expression of selected genes related to neutral lipid/cholesterol synthesis, lipid oxidation, glucose homeostasis, and ER stress. Mice were also evaluated by indirect calorimetry, ipGTT, and plasma lipid profiling. To identify very short term responses to changes in expression of *Ildr2*, we transduced mouse primary hepatocytes with the ADKD and ADOX constructs and analyzed responses at 24 hr p.t.

### Ildr2 does not Cross-regulate with Ildr1 or Ildr3

Although the apparent lack of cellular colocalization of ILDR2 with other molecules of this family makes it unlikely that ILDR2 interacts directly with them, its functions could be mediated through secondary genetic effects. To test this possibility we analyzed transcription levels among the *ildr* genes in primary B6 mouse hepatocytes transduced with siRNAs specific to each gene (**Table 1**). Whereas siRNA specific to *Ildr2* almost completely suppressed its own expression, it reduced expression of *Ildr1* by only 3% and *Ildr3* by 27%, with little effect on *Ildr2* of knockdown of either *Ildr1* or *Ildr3*. These results indicate that expression levels of *Ildr* genes do not significantly cross-regulate.

**Table 1:** Relative expression of *Ildr*-family genes transduced with *Ildr*-siRNAs.

	*Ildr1* siRNA (n = 3)		*Ildr2* siRNA (n = 10)		*Ildr3* siRNA (n = 3)	
Gene	Relative Expression	P-value	Relative Expression	P-value	Relative Expression	P-value
*Ildr1*	0.14	1.2 E-03	0.97	NS	ND	NA
*Ildr2*	0.97	NS	0.03	6.5 E-06	1.28	3.3 E-03
*Ildr3*	ND	n/a	0.73	8.0 E-05	0.03	3.4 E-03

Genes of the *Ildr*-family do not significantly cross-regulate. Data for effects of *Ildr1* and *Ildr3* siRNAs were determined by qPCR. Data for effects of *Ildr2* siRNA are from microarray. Levels of mRNA are normalized to the 36B4 ribosomal housekeeping gene, expressed relative to levels of each gene in control cells transduced with a scrambled, non-specific siRNA sequence. n/a: not applicable; ND: not done; NS: not significant.

### Changes in Ildr2 Expression Affect Liver Morphology and Histology

**Figure 3:**
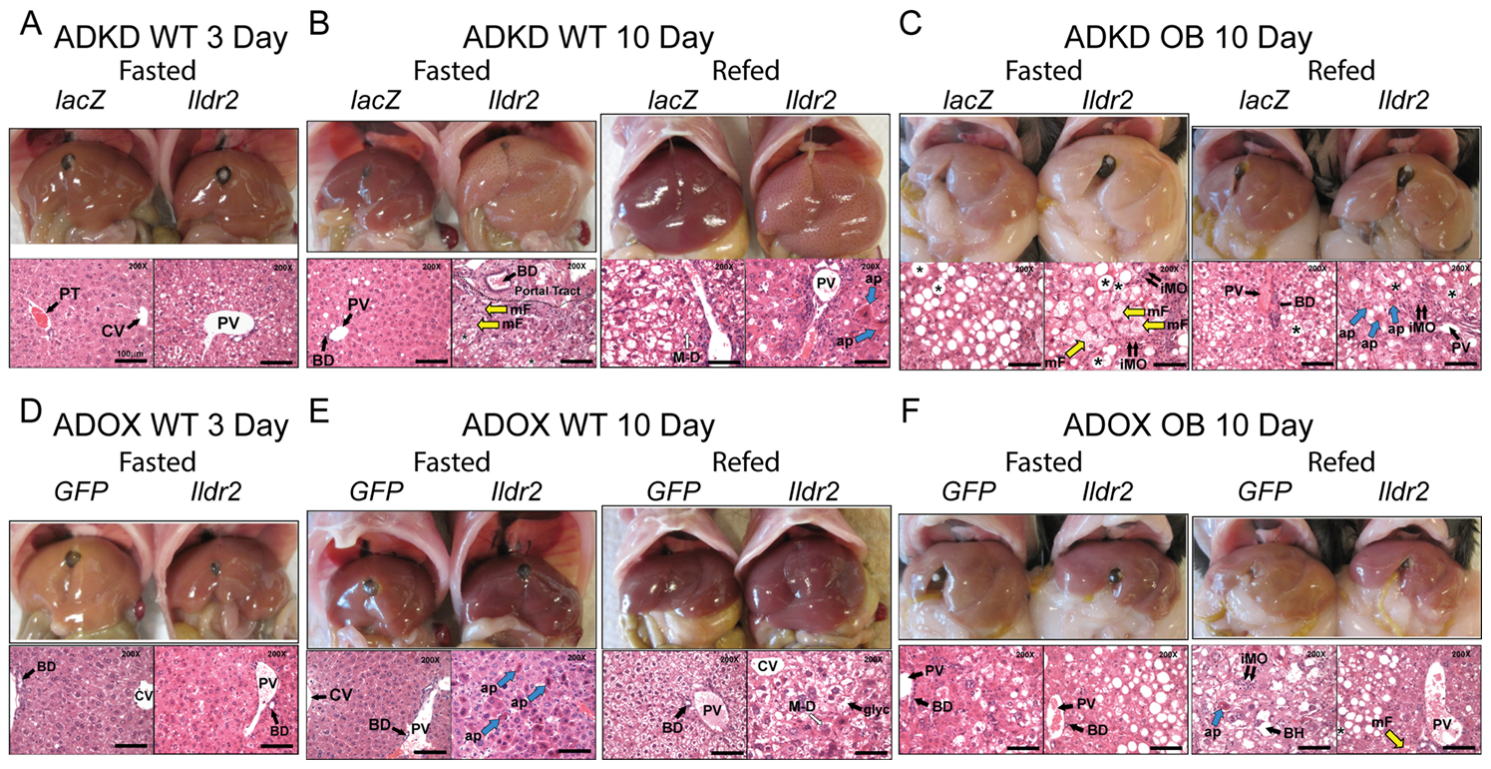
Liver morphology and histology in ADKD and ADOX WT and OB mice. Chow-fed, 10-week-old B6 males were sacrificed after 24-hr fast (Fasted) or following a 24-hr fast and 12-hr refeeding (Refed). Liver morphology is shown in the upper panels and hematoxylin and eosin staining of representative sections is shown in the lower panels at 200X magnification (scale is 100 µm). Asterisk (*) identifies large droplet, macrovesicular lipid vacuoles, particularly evident in Ob sections; large open arrows (M-D) denote intra-hepatocellular Mallory-Denk-like eosinophilic material; open yellow arrows (mF) denote small droplet, microvesicular fat within hepatocytes; short double black arrows (iMO) indicate mononuclear inflammatory cells, consistent with lymphocytes; large blue arrows (ap) indicate apoptotic hepatocytes; (glyc) identifies a “clear”-appearing hepatocyte with increased glycogen content (e.g., ADOX WT 10d Refed); Portal Tract (or PT) is above the hatched line in ADKD WT 10d Fasted); (CV) is Central Vein; (PV) is Portal Vein; (BD) is Bile Duct. (A) Wild-type mice, 3 days p.t. with adenovirus knockdown vectors expressing RNAi for *lacZ* or *Ildr2* (B) Wild-type mice, 10 days p.t. with adenovirus knockdown vectors expressing RNAi for *lacZ* or *Ildr2* (C) *ob/ob* mice, 10 days p.t. with adenovirus knockdown vectors expressing RNAi for *lacZ* or *Ildr2* (D) Wild-type mice, 3 days p.t. with adenovirus vector over-expressing *GFP* or *Ildr2*; there is no significant steatosis or inflammation (E) Wild-type mice, 10 days p.t. with adenovirus vector over-expressing *GFP* or *Ildr2* (F) *ob/ob* mice, 10 days p.t. with adenovirus vector over-expressing *GFP* or *Ildr2*. As described in the text, increased apoptosis without inflammation is consistent with a primary role for ILDR2 in ER stress responses.

Control WT livers (*lacZ*) were normal in size and appearance in WT animals at 3 and 10 days p.t. but, as expected, were enlarged and grossly steatotic in OB animals (**Figure 3**). The ADKD livers (WT and OB) were enlarged and grossly steatotic, whereas the ADOX WT livers were generally normal in appearance and size as were, remarkably, the ADOX OB livers.

Control WT livers were histologically normal with the exception of occasional mild lipid vesiculation and attendant monocytic infiltration, presumably due to adenovirus transduction *per se*. As anticipated, OB control livers showed extensive large vacuolization with minimal focal lobular lymphocytic infiltration [21]. Livers of ADKD WT mice at 3 days p.t. (**Figure 3A**) showed mildly increased periportal vacuolization, modest mononuclear infiltration, occasional apoptosis and autophagy. By 10 days p.t. (**Figure 3B**), histologic changes were striking: smaller lipid vesicles in the periportal region progressed to larger droplets at a distance from the portal tract, with ballooning of hepatocytes, autophagy, apoptosis and periportal monocytic inflammation. Some cells showed clumped pink intermediate filaments resembling human Mallory-Denk bodies in steatohepatitis, where they signify hepatocellular oxidative stress [22]. In the fed ADKD animals, increased apoptosis and inflammation were apparent in the context of a preponderance of large droplet fat vesicles. Lobular inflammation reminiscent of human non-alcoholic steatohepatitis was also seen. The livers of ADKD OB mice at 10 days p.t. (**Figure 3C**) displayed extensive lipid deposition, with microvesiculation accompanied by severe monocytic infiltration, and areas of fibrosis in some animals.

In ADOX WT animals, phenotypic effects were generally modest. At 3 days p.t. (**Figure 3D**), livers showed mild, small droplet steatosis, but by 10 days p.t. (**Figure 3E**), there were areas of increased apoptosis with minimal lipid deposition or inflammation, consistent with a primary effect on ER stress-related responses. In fed animals, glycogen deposition was greatly increased. ADOX OB animals at 10 days p.t. (**Figure 3F**) showed substantial reduction in the severity of steatosis (mostly medium and large droplet) with virtually no inflammation or apoptosis. These changes represented a striking “rescue” of the histology seen in the OB control and KD animals.

These gross effects and microscopic characteristics indicate the importance of *Ildr2* in hepatic lipid homeostasis, with reduced expression causing lipid accumulation and overexpression acting to reduce this excess in OB livers. These effects generally increased in severity between 3 and 10 days p.t. Potential mechanisms for these effects and their molecular and physiological consequences were investigated.

### Liver and Plasma Chemistry

Hepatic triglyceride (TG) content was generally consistent with the histological effects of ADKD (**Tables 2** **,** **3** **, and** **4**) and ADOX (**Tables 5** **,** **6** **, and** **7**), whereas plasma TG, FFA, and glucose/insulin-related measurements were minimally affected. In contrast, hepatic and plasma cholesterol were greatly increased in refed 10 day ADKD animals. These phenotypes are described in more detail below.

**Table 2:** Liver and plasma chemistries of ADKD WT mice at 3 days p.t.

ADKD WT 3 D			
Fasted			
Phenotype (n)	*lacZ* (10)	*Ildr2* (10)	P-value
Body weight (g)	25.9±0.5	25.7±0.8	0.831
Liver weight (g)	1.2±0.0	1.2±0.0	0.772
Hepatic TG (mg/g Liver)	53.7±5.1	69.2±3.1	0.018
Hepatic TCH (mg/g Liver)	6.7±0.1	7.2±0.3	0.268
Hepatic glycogen (mg/g Liver)	8.6±2.0	20.2±2.9	0.008
Plasma glucose (mg/dL)	182±10	216±16	0.096
Plasma insulin (µg/L)	0.15±0.04	0.26±0.06	0.190
Plasma TG (mg/dL)	156.8±29.7	157.3±20.0	0.989
Plasma TCH (mg/dL)	67.4±1.4	70.8±2.4	0.250
Plasma FFA (mEq/L)	1.27±0.03	1.29±0.02	0.564
Plasma ALT (mU/L)	134.3±10.3	126.2±7.8	0.064
Plasma AST (mU/L)	125.5±8.0	126.8±7.3	0.707
HOMA2-IR	0.59±0.17	1.11±0.33	0.178
HOMA2-B (%)	14.3±3.3	16.5±2.8	0.598

Mice were chow-fed, 10-week-old B6 (WT) males, intravenously injected with ADKD vectors expressing RNAi for *lacZ* or *Ildr2*. Measurements were taken at 3 days p.t. (following a 12-hr fast). n = number of animals in each study. Data shown are mean ± SEM; *P* values were calculated by 2-tailed *t* test. FFA, free fatty acids; ALT, alanine aminotransferase; AST, aspartate aminotransferase; HOMA2-IR, homeostasis model assessment-estimated insulin resistance; HOMA-2-B (%), homeostasis model assessment-β-cell function.

**Table 3:** Liver and plasma chemistries of ADKD WT mice at 10 days p.t.

ADKD WT 10 D						
	Fasted			Refed		
Phenotype (n)	*lacZ* (5)	*Ildr2* (6)	P-value	*lacZ* (5)	*Ildr2 (6)*	P-value
Body weight (g)	25.1	27.5	0.084	27.5	27.5	0.983
Liver weight (g)	1.3	2.1	0.000	2.1	2.5	0.096
Hepatic TG (mg/g Liver)	46.4±2.9	88.6±6.5	0.001	18.6±2.8	76.9±7.0	0.000
Hepatic TCH (mg/g Liver)	9.8±0.6	18.0±1.7	0.005	7.6±0.4	14.8±1.4	0.003
Hepatic glycogen (mg/g Liver)	1.9±0.2	3.2±0.5	0.039	58.0±3.6	37.1±2.1	0.007
Plasma glucose (mg/dL)	81±7	86±3	0.619	187±5	153±8	0.009
Plasma insulin (µg/L)	0.26±0.10	0.25±0.02	0.903	4.91±0.75	5.99±1.68	0.576
Plasma TG (mg/dL)	114.2±19.0	138.0±17.0	0.362	203.8±22.9	243.0±27.0	0.275
Plasma TCH (mg/dL)	131.7±4.0	254.0±24.8	0.003	135.5±8.8	313.3±32.4	0.001
Plasma FFA (mEq/L)	1.52±0.06	1.25±0.13	0.093	1.05±0.18	0.72±0.03	0.150
Plasma ALT (mU/L)	44.5±6.2	51.1±9.3	0.598	46.6±3.0	48.3±6.0	0.812
Plasma AST (mU/L)	46.5±6.8	48.8±9.2	0.852	49.6±3.7	47.5±6.5	0.797
HOMA2-IR	0.85±0.35	0.80±0.09	0.901	n/a	n/a	n/a
HOMA2-B (%)	94.4±22.2	91.0±9.0	0.989	n/a	n/a	n/a

Mice were chow-fed, 10-week-old B6 (WT) males, intravenously injected with ADKD vectors expressing RNAi for *lacZ* or *Ildr2*. Measurements were taken at 10 days p.t. (following either a 24-hr fast “Fasted” or following a 24-hr fast and 12-hr refeeding “Refed”). n = number of animals in each study. n/a: not applicable. Data shown are mean ± SEM; *P* values were calculated by 2-tailed *t* test. TG, triglycerides; TCH, total cholesterol; FFA, free fatty acids; ALT, alanine aminotransferase; AST, aspartate aminotransferase; HOMA2-IR, homeostasis model assessment-estimated insulin resistance; HOMA-2-B (%), homeostasis model assessment-β- cell function. In contemporaneous experiments in our laboratory, B6 mice fasted for 12 hr and not treated with adenovirus had plasma ALT of 86.0 mU/L and AST of 94.6 mU/L.

**Table 4:** Liver and plasma chemistries of ADKD OB mice at 10 days p.t.

ADKD OB 10 D						
	Fasted			Refed		
Phenotype (n)	*lacZ* (3)	*Ildr2* (4)	P-value	*lacZ* (3)	*Ildr2* (4)	P-value
Body weight (g)	46.7±0.4	46.8±0.9	0.961	46.4±3.1	47.3±1.9	0.845
Liver weight (g)	4.2±0.5	5.8±0.3	0.012	3.4±0.1	6.4±0.7	0.047
Hepatic TG (mg/g Liver)	77.5±4.0	113.7±6.8	0.037	77.0±1.0	118.0±7.3	0.041
Hepatic TCH (mg/g Liver)	10.6±1.5	16.5±3.4	0.189	8.9±0.7	16.9±3.6	0.203
Hepatic glycogen (mg/g Liver)	43.0±0.4	14.6±3.8	0.004	44.2±1.9	5.8±1.3	0.001
Plasma glucose (mg/dL)	175±29	116±20	0.215	379±79	214±28	0.233
Plasma insulin (µg/L)	18.1±10.1	27.2±19.5	0.707	77.8±33.1	103.5±5.3	0.576
Plasma TG (mg/dL)	97.6±28.0	133.5±16.1	0.395	121.0±24.5	195.4±24.2	0.135
Plasma TCH (mg/dL)	110.3±2.5	104.0±2.5	0.173	112.6±9.7	133.7±8.5	0.231
Plasma FFA (mEq/L)	1.23±0.01	1.71±0.08	0.012	1.31±0.07	2.44±0.16	0.013
Plasma ALT (mU/L)	93.2±4.0	107.2±4.1	0.006	105.9±9.6	126.8±25.3	0.274
Plasma AST (mU/L)	88.9±2.3	110.0±11.3	0.036	93.4±10.6	115.8±37.1	0.421

Mice were chow-fed, 10-week-old B6.V-Lep^ob/J^ (OB) males, intravenously injected with ADKD vectors expressing RNAi for *lacZ* or *Ildr2*. Measurements were taken at 10 days p.t. (following either a 24-hr fast “Fasted” or following a 24- hr fast and 12-hr refeeding “Refed”). n = number of animals in each study. Data shown are mean ± SEM; *P* values were calculated by 2-tailed *t* test. TG, triglycerides; TCH, total cholesterol; FFA, free fatty acids; ALT, alanine aminotransferase; AST, aspartate aminotransferase.

**Table 5:** Liver and plasma chemistries of ADOX WT mice at 3 days p.t.

ADOX WT 3 D			
Fasted			
Phenotype (n)	*GFP* (9)	*Ildr2* (9)	P- value
Body weight (g)	25.1±0.7	24.3±0.6	0.439
Liver weight (g)	1.2±0.0	1.0±0.0	0.004
Hepatic TG (mg/g Liver)	93.0±11.9	124.3±9.6	0.073
Hepatic TCH (mg/g Liver)	10.7±1.2	15.6±1.6	0.038
Hepatic glycogen (mg/g Liver)	14.6±2.2	6.2±2.1	0.009
Plasma glucose (mg/dL)	159±6	166±13	0.637
Plasma insulin (µg/L)	0.15±0.04	0.21±0.04	0.316
Plasma TG (mg/dL)	127.3±21.9	153.3±14.2	0.338
Plasma TCH (mg/dL)	65.3±0.9	72.1±2.8	0.052
Plasma FFA (mEq/L)	1.28±0.03	1.35±0.09	0.502
Plasma ALT (mU/L)	79.5±15.1	99.4±13.1	0.008
Plasma AST (mU/L)	78.0±20.5	93.3±13.0	0.089
HOMA2-IR	0.56±0.16	0.79±0.15	0.347
HOMA2-B (%)	17.7±3.8	23.5±4.7	0.302

Mice were chow-fed, 10-week-old B6 (WT) males, intravenously injected with ADOX vectors expressing *GFP* or *Ildr2*. Measurements were taken at 3 days p.t. (following a 12-hr fast). n = number of animals in each study. Data shown are mean ± SEM; *P* values were calculated by 2-tailed *t* test. TG, triglycerides; TCH, total cholesterol; FFA, free fatty acids; ALT, alanine aminotransferase; AST, aspartate aminotransferase; HOMA2-IR, homeostasis model assessment-estimated insulin resistance; HOMA-2-B (%), homeostasis model assessment- β-cell function.

**Table 6:** Liver and plasma chemistries of ADOX WT mice at 10 days p.t.

ADOX WT 10 D						
	Fasted			Refed		
Phenotype (n)	*GFP* (5)	*Ildr2* (6)	P-value	*GFP* (5)	*Ildr2* (6)	P-value
Body weight (g)	24.1	23.2	0.607	27.5	26.0	0.112
Liver weight (g)	1.0	1.3	0.011	1.7	2.2	0.030
Hepatic TG (mg/g Liver)	64.9±6.9	33.0±4.7	0.004	31.3±5.5	20.0±5.6	0.191
Hepatic TCH (mg/g Liver)	11.4±0.8	7.4±0.6	0.005	6.8±0.7	5.4±0.9	0.272
Hepatic glycogen (mg/g Liver)	1.1±0.3	4.0±0.9	0.033	63.0±1.3	71.2±0.4	0.048
Plasma glucose (mg/dL)	105±4	92±4	0.056	211±4	155±3	0.001
Plasma insulin (µg/L)	0.34±0.09	0.34±0.07	0.988	5.67±0.09	4.95±0.07	0.560
Plasma TG (mg/dL)	57.6±2.5	98±9.6	0.005	192.8±23.0	246.1±22.4	0.117
Plasma TCH (mg/dL)	114.6±8.9	123.9±11.6	0.563	125.7±2.3	145.7±1.2	0.005
Plasma FFA (mEq/L)	1.25±0.16	1.70±0.19	0.148	0.65±0.18	0.82±0.10	0.470
Plasma ALT (mU/L)	66.9±5.1	32.1±0.3	0.021	72.2±6.3	41.1±1.9	0.042
Plasma AST (mU/L)	65.6±3.7	31.4±0.5	0.012	68.0±6.2	41.1±2.7	0.028
HOMA2-IR	1.36±0.25	1.10±0.19	0.435	n/a	n/a	n/a
HOMA2-B (%)	86.0±14.8	100.0±18.8	0.570	n/a	n/a	n/a

Mice were chow-fed, 10-week-old B6 (WT) males, intravenously injected with ADOX vectors expressing *GFP* or *Ildr2*. Measurements were taken at 10 days p.t. (following either a 24-hr fast “Fasted” or following a 24-hr fast and 12-hr refeeding “Refed”). n = number of animals in each study. n/a: not applicable. Data shown are mean ± SEM; *P* values were calculated by 2-tailed *t* test. TG, triglycerides; TCH, total cholesterol; FFA, free fatty acids; ALT, alanine aminotransferase; AST, aspartate aminotransferase; HOMA2-IR, homeostasis model assessment-estimated insulin resistance; HOMA-2-B (%), homeostasis model assessment-β-cell function.

**Table 7:** Liver and plasma chemistries of ADOX OB mice at 10 days p.t.

ADOX OB 10 D						
	Fasted			Refed		
Phenotype (n)	*GFP* (4)	*Ildr2* (4)	P-value	*GFP* (4)	*Ildr2* (4)	P-value
Body weight (g)	45.1±1.4	48.2±0.6	0.120	46.9±1.1	43.7±0.8	0.066
Liver weight (g)	3.4±0.2	3.9±0.3	0.321	4.6±0.3	3.4±0.1	0.042
Hepatic TG (mg/g Liver)	49.5±5.7	27.1±3.0	0.019	56.4±4.4	32.3±4.0	0.007
Hepatic TCH (mg/g Liver)	7.7±0.6	5.6±0.2	0.038	6.4±0.2	5.1±0.29	0.015
Hepatic glycogen (mg/g Liver)	50.4±4.3	53.5±3.7	0.606	62.8±101.6	61.3±103.6	0.689
Plasma glucose (mg/dL)	151±10	174±24	0.420	224±29	263±20	0.349
Plasma insulin (µg/L)	13.6±4.4	24.5±2.5	0.168	73.9±19.1	89.3±4.0	0.489
Plasma TG (mg/dL)	94.3±27.2	129.8±43.4	0.538	137.6±18.3	135.4±11.7	0.925
Plasma TCH (mg/dL)	134.0±3.5	129.0±3.9	0.383	143.1±4.8	130.9±2.6	0.086
Plasma FFA (mEq/L)	1.66±0.09	1.46±0.05	0.119	2.25±0.29	1.76±0.12	0.206
Plasma ALT (mU/L)	115.1±18.4	109.7±15.7	0.668	108.0±5.4	100.1±15.3	0.386
Plasma AST (mU/L)	106.1±8.8	101.7±13.8	0.611	102.7±12.7	98.7±17.0	0.718

Mice were chow-fed, 10-week-old B6.V-Lep^ob/J^ (OB) males, intravenously injected with ADOX vectors expressing *GFP* or *Ildr2*. Measurements were taken at 10 days p.t. (following either a 24-hr fast “Fasted” or following a 24 hr fast and 12 hr refeeding “Refed”). n = number of animals in each study. Data shown are mean ± SEM; *P* values were calculated by 2-tailed *t* test. TG, triglycerides; TCH, total cholesterol; FFA, free fatty acids; ALT, alanine aminotransferase; AST, aspartate aminotransferase.

At 3 days p.t. in ADKD WT animals (**Table 2**), body weight, liver weight and liver total cholesterol (TCH) content were unaffected, as were circulating concentrations of glucose, insulin, TG, TCH, and FFA. However, hepatic TG and glycogen content were significantly increased. Estimates of insulin resistance by HOMA2-IR and β-cell function by HOMA-2B% were unaffected, as were glucose excursions during IPGTT (see below). At 10 days p.t. in ADKD WT animals (**Table 3**), hepatic and circulating TCH and hepatic TG were increased while circulating TG was unchanged. Again, HOMA measurements were unaffected. In ADKD OB mice at 10 days p.t. (**Table 4**), - starting from higher baselines (as expected vs. WT animals) [21] - liver weight, TG and TCH content increased, and glycogen content decreased. Circulating concentrations of glucose, insulin, TG, TCH, and IPGTT were unaffected; circulating FFA concentrations were increased.

In ADOX WT animals at 3 days p.t. (**Table 5**), TG and TCH concentrations per unit wet weight of liver were higher (and glycogen lower) than in control mice. Plasma lipids were unaffected by ADOX. At 10 days p.t. in ADOX WT mice (**Table 6**), liver TG was lower, and glycogen content higher than in controls. Plasma TG and TCH trended higher in the ADOX animals. At 10 days p.t. in ADOX OB mice (**Table 7**)**,** liver TG and TCH content were reduced without significant changes in circulating glucose, TG, TCH or FFA. Hepatic glycogen per gram wet weight was unaffected but, given the considerable reduction of TG, was probably decreased per unit liver nitrogen. Measurements of blood ALT and AST enzyme levels in these animals indicate that toxic effects of the adenoviral transductions on hepatocyte integrity were minimal.

### Analysis of *in vivo* Lipoprotein Production and Clearance

**Figure 4:**
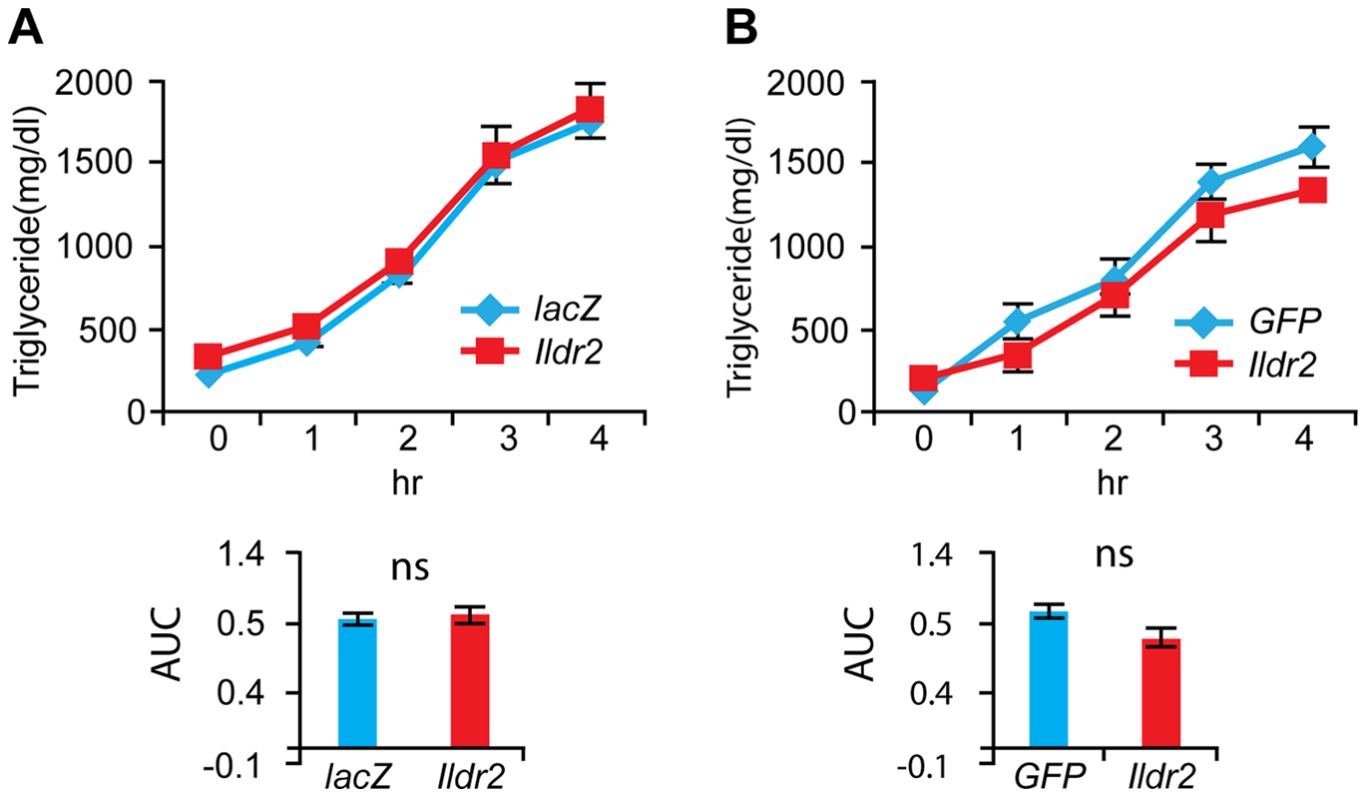
TG secretion analysis in ADKD and ADOX WT mice. Chow-fed, 10-week-old B6 (WT) males were intravenously injected with ADKD or ADOX vectors expressing RNAi for *lacZ* or *Ildr2*. At 7 days p.t., following a 16 hr fast, mice were intravenously injected with 15% Triton WR1339 at a dose of 500 mg/kg. Plasma (from 100 ul of blood) was collected hourly for 4 hr and TG measured. (A) Wild-type mice, 7 days p.t. with adenovirus knockdown vector expressing RNAi for *lacZ* or *Ildr2*; (B) Wild-type mice, 7 days post- transduction with adenovirus vector over-expressing *GFP* or *Ildr2.* AUC: area under the curve. Insignificant differences by AUC analysis show that hepatic lipoprotein secretion is unaffected by Triton WR1339 administration in ADKD and ADOX mice.

The increase in hepatic TG and TCH in the ADKD mice could reflect: 1. increased assembly/reduced secretion of lipoproteins; 2. increased synthesis/decreased oxidation of TG; 3. increased synthesis/decreased disposal of cholesterol. Detergents such as Triton WR1339 block clearance of TG in circulating lipoproteins by inhibiting lipoprotein lipase (LPL)-mediated lipolysis of circulating TG-rich lipoproteins [23]. When LPL is inhibited, changes in circulating concentrations of lipoprotein species reflect hepatic secretion rates of very low-density lipoprotein (VLDL). We measured concentrations of plasma TG following LPL inhibition with Triton WR1339 in 10-week-old chow fed ADKD and ADOX WT mice at 7 days p.t. (**Figure 4**). Area under the curve (AUC) analysis of hepatic lipoprotein secretion shows no significant difference in either ADKD (**Figure 4A**) or ADOX mice (**Figure 4B**). These results suggest that in ADKD mice, the increased hepatic lipids did not stimulate increased VLDL secretion and that, consequently, the increased plasma lipids, notably TCH, reflected reduced hepatic lipoprotein clearance. Likewise, the decreased hepatic lipids in ADOX mice were not due to increased VLDL secretion. Increased hepatic lipid synthesis in ADKD mice was not coupled to secretion or decreased hepatic fatty acid oxidation. In contrast, ADOX mice could have had decreased hepatic lipid synthesis or increased fatty acid oxidation. Finally, it is interesting to note that in these animals, glucose tolerance was normal despite severe hepatic steatosis [24].

### Steady-state Lipoprotein Analysis

**Figure 5:**
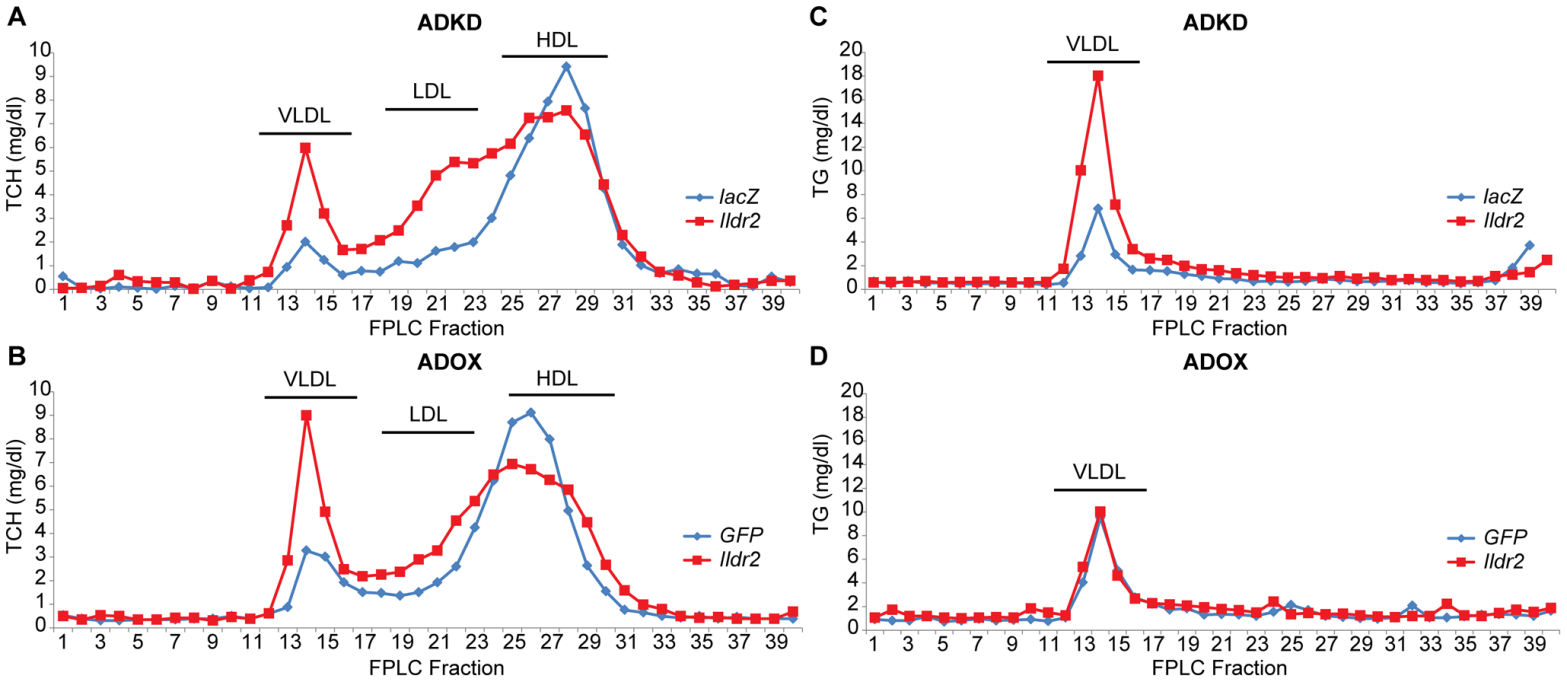
FPLC analysis of plasma lipoprotein fractions in ADKD and ADOX WT mice. At 7 days p.t. with either ADKD or ADOX vectors, plasma from 6 wild-type mice was collected, pooled and TCH and TG profiles were analyzed by FPLC using Sepharose 6 Fast Flow columns. HDL, high-density lipoprotein; LDL, low-density lipoprotein; VLDL, very low-density lipoprotein. (A) TCH profile in wild-type mice, 7 days p.t. with adenovirus knockdown vector expressing RNAi for *lacZ* or *Ildr2*; (B) TCH profile in wild-type mice, 7 days p.t. with adenovirus vector over-expressing *GFP* or *Ildr2*; (C) TG profile in wild-type mice, 7 days after adenovirus knockdown vector expressing RNAi for *lacZ* or *Ildr2*; (D) TG profile in wild-type mice, 7 days p.t. with adenovirus vector over-expressing *GFP* or *Ildr2*. These experiments show an increase in plasma TG (as VLDL) in ADKD mice but not in ADOX mice. TCH shifts in ADKD mice from HDL to LDL and VLDL, while in ADOX mice the decrease in HDL is accompanied by an increase in VLDL only.

Based upon the striking changes in hepatic lipid content without evidence of change in lipoprotein export, we were interested in qualitative and quantitative changes in circulating lipoproteins in response to transient manipulations of *Ildr2* expression. We were particularly interested in determining if the dramatic increase in TCH in the ADKD mice represented increased TCH in VLDL or LDL, or decreased TCH in high-density lipoprotein (HDL). In analysis by fast protein liquid chromatography (FPLC) of fasted plasma obtained prior to the Triton study (**Figure 5** **)**, VLDL cholesterol (fractions 12–16) and IDL/LDL cholesterol (fractions 17–23) were clearly higher, and HDL cholesterol (fractions 24–30) was ∼20% lower in the ADKD animals (**Figure 5A, 5C**). In ADOX animals (**Figure 5B, 5D**), VLDL was similarly increased, LDL was not altered, and HDL was also slightly reduced. These results are consistent with the data reported in **Tables 2** **,** **3** **,** **4** **,** **5** **,** **6** **, and** **7**, in which ADKD animals had higher absolute circulating TG than ADOX animals and where WT fasted and refed ADKD mice exhibited increases in plasma TCH and TG vs. controls, whereas WT ADOX mice exhibited more moderate changes.

### Hepatic Gene-expression Signatures

**Figure 6:**
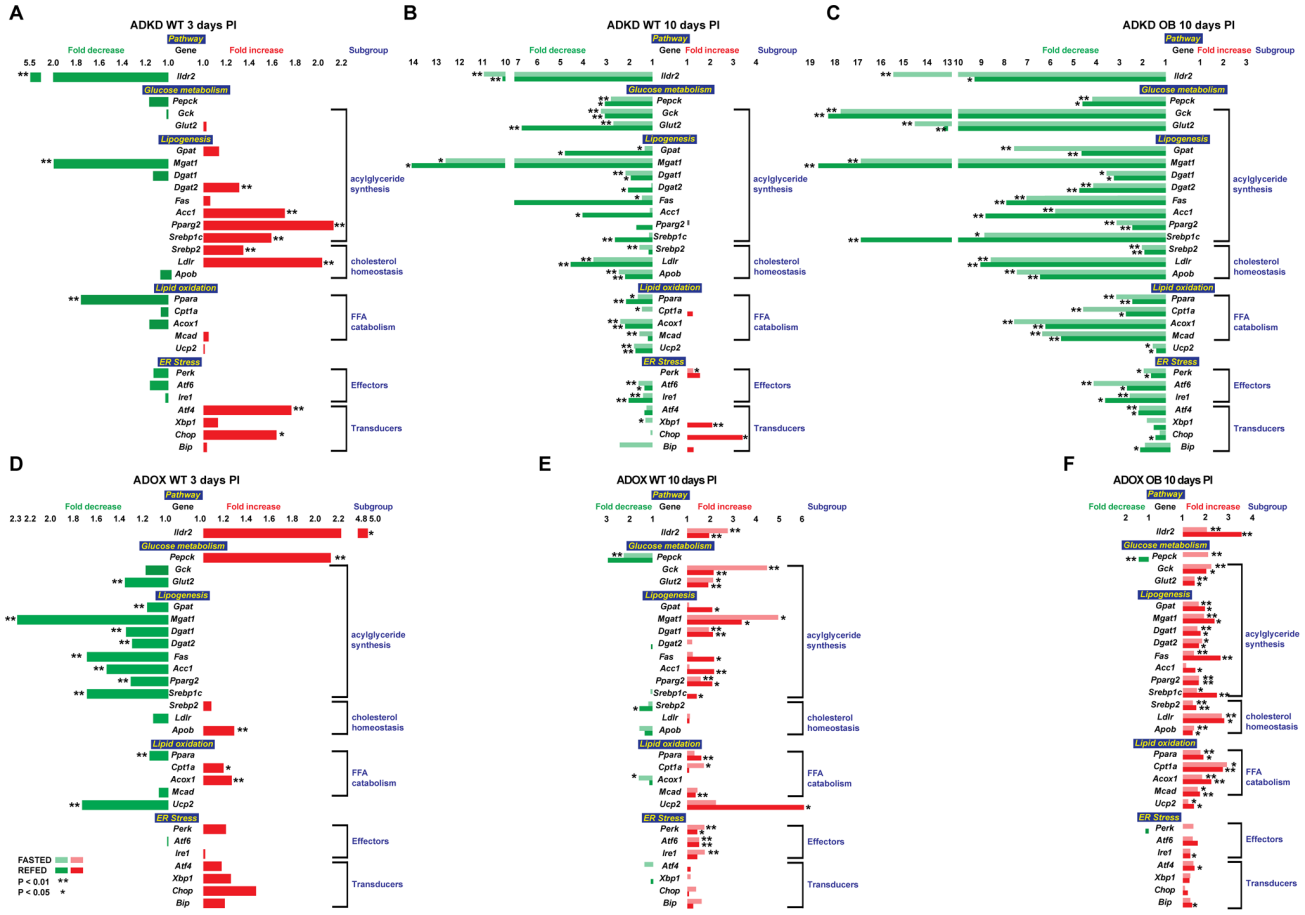
Relative expression of selected genes in ADKD and ADOX WT and OB mice. 10-week-old B6 male mice were chow-fed, intravenously injected with ADKD and ADOX vectors and sacrificed at 3 days p.t, following a 12-hr fast. Expression levels were determined by qPCR normalized to expression levels of the 36B4 housekeeping gene. Fold changes are relative to the *GFP* control in the same state as the *Ildr2* (either fasted or refed compared to fasted or refed). * indicates p<0.05; ** indicates p<0.01 (2 tailed t-test). (A) Expression in wild-type mice, 3 days p.t. with adenovirus knockdown vector expressing RNAi for *lacZ* or *Ildr2*; (B) Expression in wild-type mice, 10 days p.t. with adenovirus knockdown vector expressing RNAi for *lacZ* or *Ildr2*; (C) Expression in wild-type mice, 10 days p.t. with adenovirus vector over-expressing *GFP* or *Ildr2*; (D) Expression in wild-type mice, 3 days p.t. with adenovirus knockdown vector expressing RNAi for *lacZ* or *Ildr2:* (E) Expression in *ob/ob* mice, 10 days p.t. with adenovirus knockdown vector expressing RNAi for *lacZ* or *Ildr2*; (F) Expression in *ob/ob* mice, 10 days p.t. with adenovirus vector over-expressing *GFP* or *Ildr2*. Changes in transcriptional profiles appear to be secondary to changes in lipid content.

To assess possible molecular bases for these changes in liver histology and lipid/glycogen chemistry with the remarkably minimal effects of these changes on systemic lipid and insulin homeostasis, we examined hepatic expression of genes related to acylglyceride, cholesterol and glucose homeostasis and ER-resident molecules mediating responses to metabolic stress (**Figure 6**).

In ADKD WT animals, at 3 days p.t. (**Figure 6A**), increases in transcript levels of genes involved in acylglyceride synthesis were consistent with increased TG content; however, at 10 days p.t. (**Figure 6B**), transcripts of genes related to acylglyceride synthesis were reduced (where *Fas* expression was especially decreased in the fed animals), as were genes related to cholesterol homeostasis and FFA catabolism. Unlike the livers at 3 days p.t., those at 10 days displayed a general suppression of transcripts of genes mediating both synthesis and oxidation of hepatic lipids. The general suppression of transcripts of genes mediating both synthesis and oxidation of hepatic lipids between days 3 and 10 suggests that the accumulation of hepatic lipids due to effects of inhibition of *Ildr2* transcription resulted in secondary suppression of the expression of these genes.

In ADKD OB animals at 10 days p.t. (**Figure 6C**), *Ildr2* knockdown greatly reduced the expression levels of all transcripts examined compared to the control (*lacZ*) animals. These effects were comparable, though more extensive and proportionately greater, than in the corresponding studies of WT animals, possibly reflecting, in part, the consequences of pre-existing hepatic steatosis.

In livers of ADOX WT mice at 3 days p.t. (**Figure 6D**), as in the ADKD livers, in the context of an increase in TCH content (and a trend towards increased TG), transcript levels of genes mediating the synthesis of these molecules were generally reduced, although transcript levels of some fatty acid oxidation genes were slightly increased. However, at 10 days p.t. (**Figure 6E**), transcript levels of genes related to glucose metabolism and acylglyceride synthesis were increased.

In livers of ADOX OB animals at 10 days p.t. (**Figure 6F**), the very high levels of lipid accumulation due to the obesity of these animals were reduced by over-expression of *Ildr2*. In association with this reduction, levels of transcripts related to neutral lipid synthesis and cholesterol are increased, suggesting “relief” of the secondary suppression imposed by lipid accumulation as mentioned above [25].

### ER Stress Pathways

The apparent fixed location of ILDR2 in the ER (see **Figure 2**) raises the possibility that the protean effects of hypomorphism for this gene might be related to a role in the mediation of ER stress responses [12]. Such a role would not be inconsistent with an independent effect on lipoprotein metabolism [26]. Accordingly, we examined transcription rates of canonical members of the ER stress response pathways [27].

In ADKD WT animals at 3 days p.t., expression of ER stress effectors *Perk*, *Atf6*, and *Ire1* was slightly reduced, while expression of transducers *Atf4* and *Chop* was increased. In general, *Ildr2* over-expression was associated with increased expression of ER stress pathway genes, while *Ildr2* knockdown was associated with decreased expression. Effects were greater at 10 days than at 3 days p.t.

**Figure 7:**
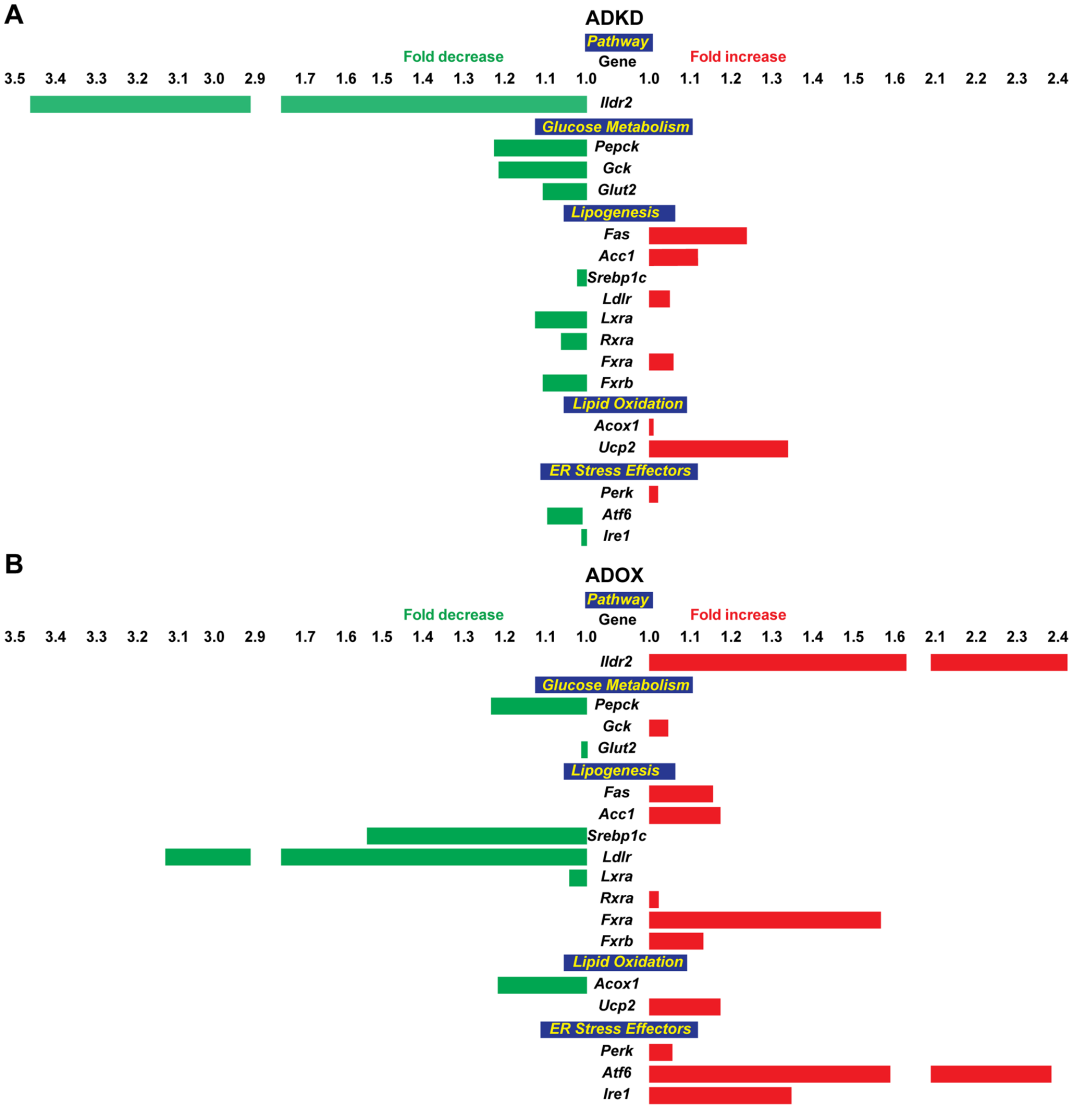
Relative expression of selected genes in ADKD and ADOX primary hepatocytes. To identify short-term effects of changes in *Ildr2* expression, hepatocytes from five 10-week-old B6 mice were extracted, pooled and plated into individual wells and exposed, in triplicate, for 24 hr to ADOX or ADKD viral vectors. RNA was extracted, transcribed into cDNA, and expression was determined by qPCR. (A) Expression in hepatocytes transduced with adenovirus knockdown vector expressing RNAi for *lacZ* or *Ildr2*; (B) Expression in hepatocytes transduced with adenovirus vector over-expressing *GFP* or *Ildr2*. These results recapitulate those seen the *in vivo* studies.

Increases in hepatocyte lipids activate ER stress pathways [28]–[30], and activation of ER stress pathways increases hepatic lipid deposition [31]–[33]. Given these reciprocal interactions, and the relatively extended time course over which these studies were conducted, it is not possible to assign causal primacy to either the effects on lipid synthesis or ER stress responses. The data are also consistent with the possibility that ILDR2 has primary effects on both processes. Experiments conducted in isolated hepatocytes (see **Figure 7**) demonstrate that *in vitro* knockdown of *Ildr2* modestly increases ER stress-related transcripts over a 48-hr period. Longer term, these responses may be exhausted [34], [35]. The apparent paradox of increased ER stress responses in both ADKD and ADOX hepatocytes may be due to the former’s reflecting the response to increased cellular lipids, and the latter to direct interactions of the ILDR2 molecule with elements of the ER stress pathways. The increase in ER stress molecules is presumably partially protective [36]–[38].

### Short-term Effects of Ildr2 Expression on Lipid Metabolism and ER Stress Pathways in Hepatocytes

ER stress can affect lipid metabolism and *vice versa* [31], [39], [40], and molecules such as XBP1 can independently affect both pathways [26]. In an effort to disarticulate – by shortening the experimental time course - possible contributions of ILDR2 to ER stress response mechanisms, we transduced C57BL/6J mouse primary hepatocytes for 24 hr with *Ildr2* ADKD and ADOX adenoviral vectors and examined cellular lipid content (**Table 8**) and expression of genes of lipid biochemical and UPR/ER stress pathways (**Figure 7**).

**Table 8:** Triglyceride and cholesterol content of ADKD and ADOX hepatocytes.

	ADKD			ADOX		
	*lacZ*	*Ildr2*	P-value	*GFP*	*Ildr2*	P-value
Triglyceride (mg/g protein)	103.1±2.3	119.4±3.4	0.019	105.5±3.9	92.7±1.3	0.056
Cholesterol (mg/g protein)	7.58±0.37	8.45±0.40	0.083	8.43±0.96	6.62±0.45	0.081

Hepatocytes from 5, 10-week-old B6 mice were extracted, pooled and plated into individual wells and exposed for 24 hr to either the ADOX or ADKD (or empty vector control) virus in triplicate. Cells were lysed and triglyceride and total cholesterol were determined.

In the ADKD cells, TG and TCH content were increased, consistent with the changes seen in the *in vivo* studies. Only slight changes were seen in the transcripts analyzed, with no indication of primacy of *Ildr2* knockdown effects on either lipid synthesis or ER stress genes (**Figure 7A**). In the ADOX cells, TG and TCH content were reduced in the context of large, reciprocal changes in both lipogenesis, where *Ldlr* and *Srebp1c* decreased, and ER stress effectors, where *Atf6* and *Ire1* increased (**Figure 7B**). The comparable magnitude of these changes makes it difficult to assign primacy, but is consistent with a role for *Ildr2* in both pathways. If these *in vitro* data at 24 hr are viewed in the context of the 3 and 10 day *in vivo* studies, it is apparent that there are strong temporal effects of responses of the ER stress pathways, and that the responses are influenced by intercurrent processes, probably lipid deposition *per se*.

### Effects of Feeding Status, Diet, and ob Genotype on Ildr2 Expression

**Figure 8:**
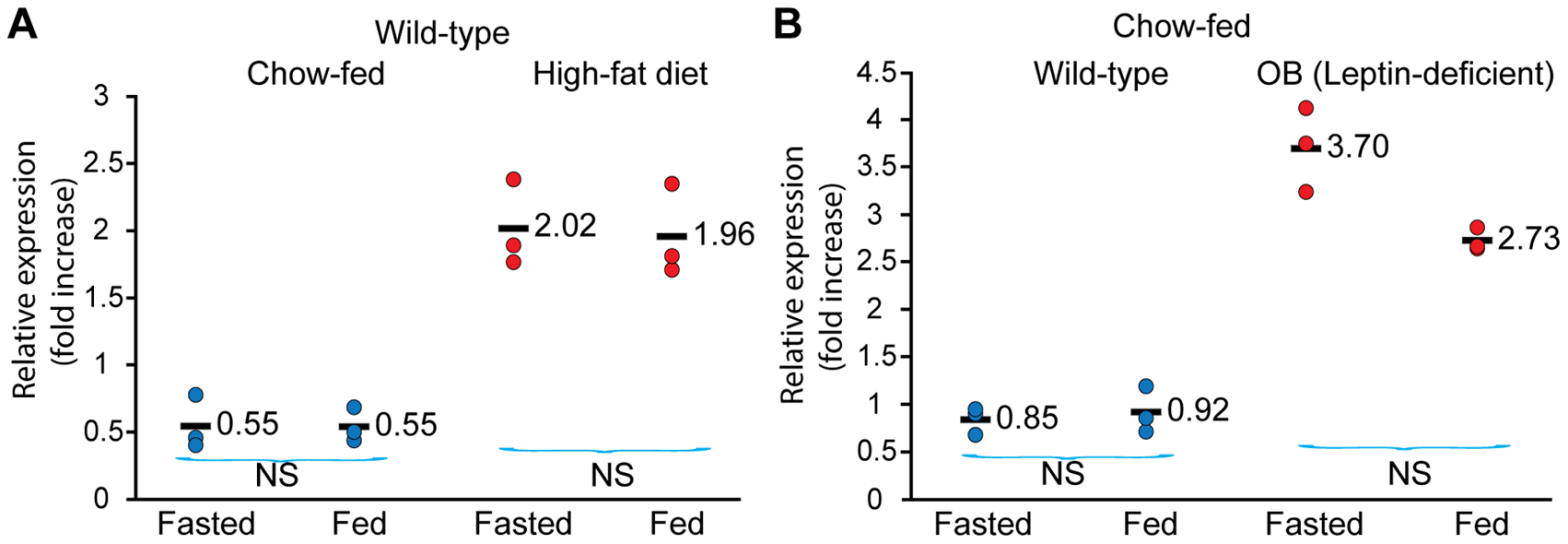
Expression of *Ildr2* in liver is increased by adiposity through high-fat diet or leptin deficiency. Expression of *Ildr2* was determined by qPCR, normalized to 36B4 in mice sacrificed after either fasting for 24 hr or after fasting for 24 hr and followed by a 12-hr refeeding period. (A) Wild type B6 mice at 6 weeks of age were fed *ad libitum* either chow or a high fat diet (60% of kcal from fat) for 3 additional months. (B) Chow-fed wild type B6 and leptin-deficient OB mice (B6.Cg-Lep*^ob^*/J) were purchased at 9 weeks and sacrificed at 10 weeks of age. Wild-type mice fed a high fat diet and genetically obese mice showed a similar (3.6 to 3.7-fold) increase in *Ildr2* liver expression compared to age- matched wild-type mice (p value <.01 ) regardless of feeding status.

Hepatic lipid homeostasis is strongly affected by fasting and refeeding and by diet [41]. To study their effects on *Ildr2*, we compared levels of *Ildr2* expression in livers of WT mice fed either chow or a high-fat diet (**Figure 8**). These results show that feeding status (the difference between fasted and refed mice) had little effect on *Ildr2* expression, whereas obesity achieved by feeding a high-fat diet, increased *Ildr2* levels by 3.6 fold (**Figure 8A**). To determine if this effect was leptin-dependent, we also analyzed *Ildr2* expression in livers of OB (leptin-deficient) mice. These mice showed a 3.7 fold increase in *Ildr2* expression compared to age-matched WT controls (**Figure 8B**). The large increases in *ildr2* expression, caused by leptin deficiency and high-fat feeding are presumably secondary – at some level - to the deposition of TG in the hepatocytes under both circumstances. Given the possible dual roles of *Ildr2*, this effect could reflect a role of *Ildr2* in ER stress responses.

### Calorimetry

**Figure 9:**
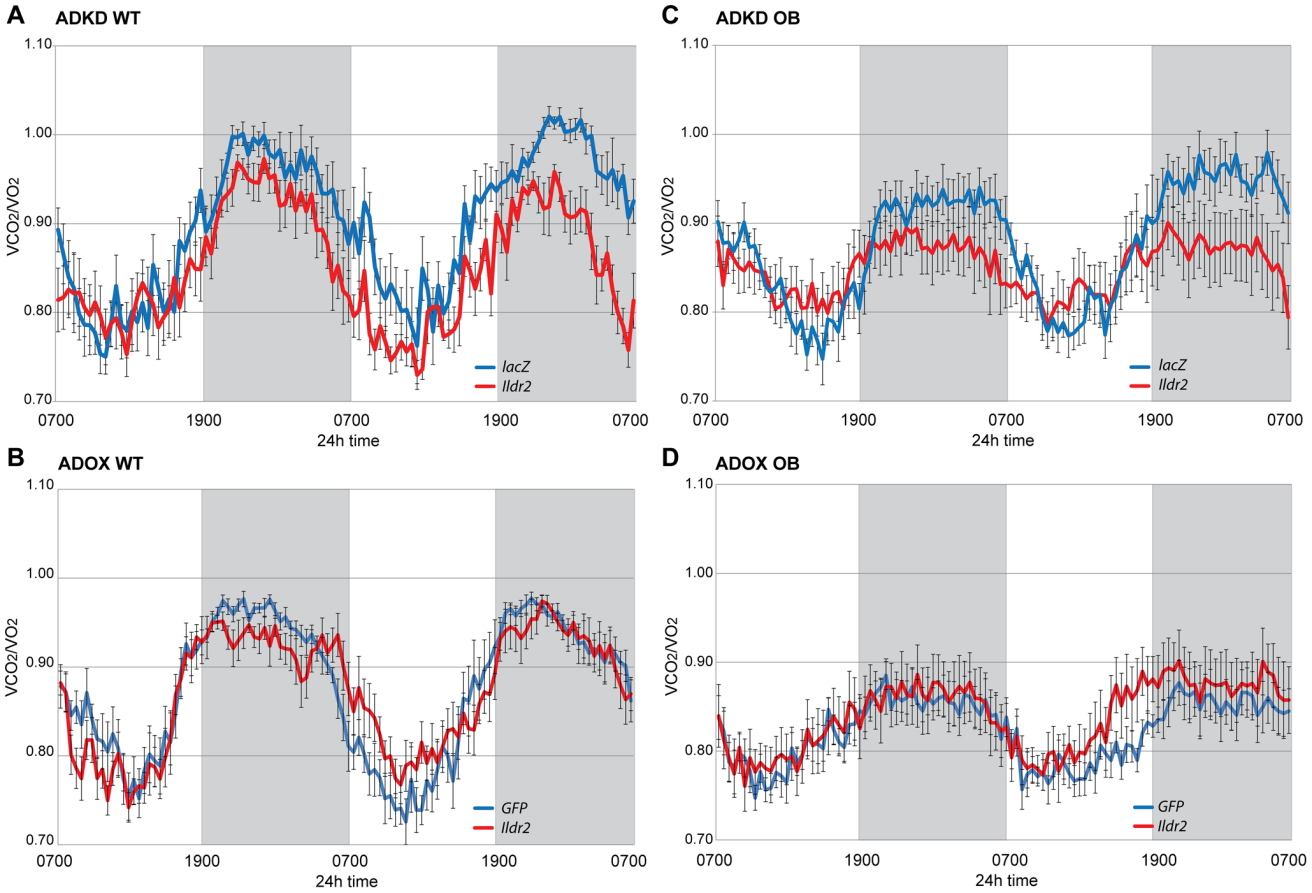
Respiratory Exchange Ratio (RER) in ADKD and ADOX WT and OB mice. Mice were chow-fed, 10-week-old B6 (WT) or B6.V-Lep^ob/J^ (OB) males, at 4 to 5 days p.t. with adenovirus knockdown vectors expressing RNAi for *lacZ* or *Ildr2* or with adenovirus vectors over- expressing *GFP* or *Ildr2*. Data shown are mean ± SEM (8 mice per group) and run in a TSE systems indirect calorimeter for 48 hr. (A) WT ADKD; (B) OB ADKD; (C) WT ADOX; (D) OB ADOX. ADKD mice show decreased RER at night, whereas ADOX mice show no differences, day or night. AUC calculations are shown in Table 9.

To determine if there were differences in energy expenditure, physical activity, or metabolic substrate use in the mice in any of the models used (ADKD and ADOX in both WT and OB), we performed indirect calorimetry (72-hr) on chow-fed, 10-week-old WT and OB males, 4 to 5 days p.t. **(** **Figure 9** **;** **Table 9** **)**.

**Table 9:** Area under the curve calculations for calorimetry in Figure 9.

	wild-type				*ob/ob*				
	ADKD		ADOX		ADKD			ADOX	
AUC	*lacZ*	*Ildr2*	*GFP*	*Ildr2*		*lacZ*	*Ildr2*	*GFP*	*Ildr2*
24-hr	43.2±0.6	40.8±0.6*	42.1±0.6	42.0±0.4		42.0±0.8	40.9±1.4	39.5±0.8	40.3±1.3
Day	20.1±0.5	19.2±0.5	19.6±0.4	19.7±0.2		19.8±0.4	20.0±0.6	19.1±0.3	19.5±0.6
Night	23.1±0.3	21.6±0.3**	22.4±0.2	22.3±0.3		22.3±0.6	20.9±0.8	20.5±0.5	20.8±0.7

* p<0.05;

** p<0.01; AUC, area under the curve.

There were no differences in rates or patterns of 24-hour energy expenditure in WT mice between knockdown and control (data not shown). However, in WT (**Figure 9A**) and OB (**Figure 9C**) ADKD mice, the nocturnal respiratory exchange ratio (RER) was 7% lower vs. controls**,** indicating that, at night, the ADKD mice preferentially oxidize fat to a greater extent than the WT mice. In WT (**Figure 9B**) and OB (**Figure 9D**) ADOX animals, the RER was not significantly different between the two groups at any time, although the OB mice had a slightly higher RER during the dark period. These data are consistent with hepatic lipid content influencing systemic fuel oxidation: higher fat content increasing fatty acid oxidation, resulting in a lower RER.

### ipGTT (intraperitoneal Glucose Tolerance Tests)

**Figure 10:**
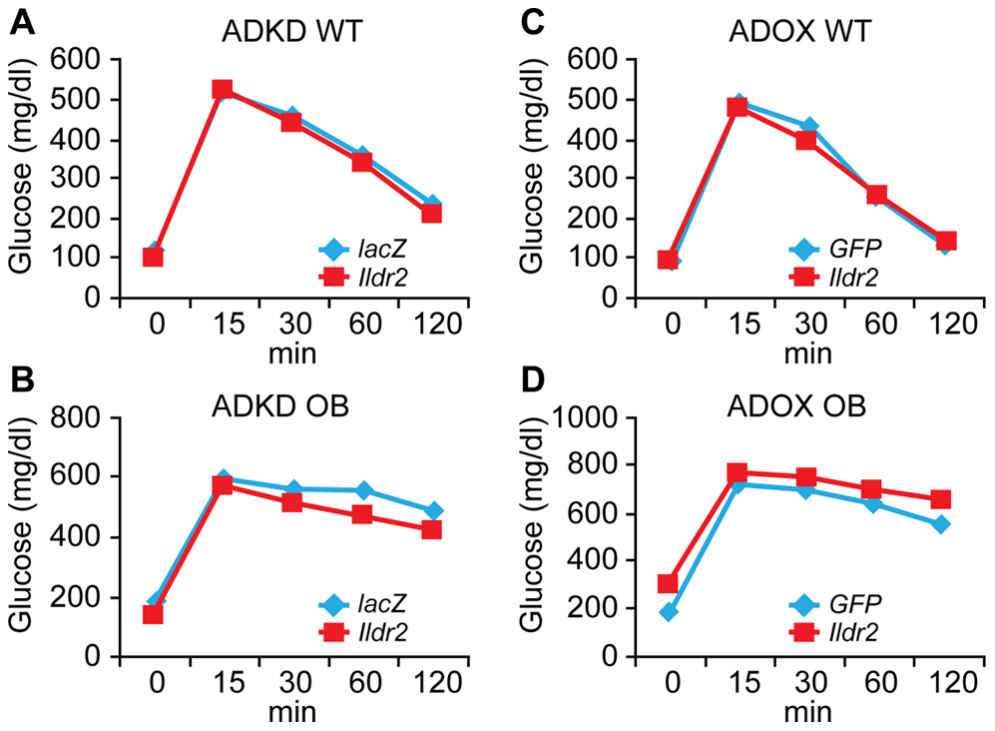
ipGTT in WT and OB mice 7 days p.t. At 7 days p.t. with adenovirus knockdown vectors expressing RNAi for *lacZ* or *Ildr2* (left) or with adenovirus vectors over-expressing *GFP* or *Ildr2* (right), the 10-week-old chow-fed male B6 mice that were used in the 10 day experiments were injected intraperitoneally after 12 hr fast with 2g/kg glucose. The mice used in this experiment are the same mice that on which indirect calorimetry was conducted on day 5 p.t. (A) WT ADKD; (B) OB ADKD; (C) WT ADOX; (D) OB ADOX. In both ADKD and ADOX animals, IPGTT was unaffected.

To assess systemic effects of changes in hepatic lipid and glycogen content on peripheral glucose homeostasis, we performed ipGTT on chow-fed, 10-week-old WT and OB males, 7 days p.t. with *Ildr2* ADKD and ADOX constructs (**Figure 10**). Surprisingly, no differences in systemic glucose tolerance were detected in ADKD or ADOX animals versus either their respective controls, or each other. Consistent with these findings, HOMA IR values based on data obtained at the time of sacrifice of ADKD and ADOX mice (at 10 days p.t.) were not significantly different (see **Table 2**). Thus, large changes in hepatic lipid content were not accompanied by changes in systemic glucose/insulin homeostasis. This finding has implications for the possible mechanism(s) underlying the effects of *Ildr2* on hepatic lipid synthesis and handling.

## Discussion

Based upon manipulation of levels of expression of *Ildr2* in liver and isolated hepatocytes using ADKD and ADOX constructs, we conclude that ILDR2 is an ER membrane protein that participates in cellular lipid synthesis and responses to ER stress. The most salient phenotype in the ADKD mice is TG accumulation, accompanied by increased hepatic and plasma cholesterol and a mix of micro- and macro-vesicular lipid droplets in periportal hepatocytes. Overexpression of *Ildr2* in *ob/ob* mice substantially rescued their hepatic steatosis, as *Ildr2* over-expressing mice had significantly decreased hepatic TG and TCH and reduced periportal vacuolar deposition.

Despite the excess lipid in the livers at 10 days p.t., transcript levels for major lipogenic and fat-oxidative genes were reduced in ADKD mice and up-regulated in ADOX mice. At 3 days p.t., several of these genes showed changes in expression in the opposite direction, suggesting that down-regulation in ADKD mice at 10 days p.t. may have been a response to excess lipid accumulation. These changes in transcriptional profiles are likely secondary to the respective increased/decreased lipid content of the hepatocytes. Also relevant in considering the molecular pathogenesis of the steatosis in the ADKD animals is the absence of major changes of circulating TG or cholesterol in these animals, their apparently normal rates of hepatic TG secretion, and the absence of significant changes in glucose or insulin homeostasis. In ADKD mice, reduced hepatic VLDL/IDL/LDL clearance and increased circulating IDL/LDL cholesterol suggests a reduction in hepatic LDL receptor-mediated clearance of those lipoproteins, consistent with reduced expression of hepatic LDL receptor [42], [43].

Lipid accumulation in the liver is commonly associated with liver and/or systemic insulin resistance and resultant hyperglycemia. Indeed, hepatic steatosis is commonly implicated as a causative factor in these phenotypes that are aspects of the metabolic syndrome [44]–[46]. However, in the ADKD animals, large changes in hepatic lipid content were not accompanied by changes in systemic glucose/insulin homeostasis [47]. Decreased lipid droplet turnover and/or enhanced traffic of newly synthesized TG from the ER to the cytoplasmic droplets might be related to the apparent absence of effect of the increased lipid deposition on glucose/insulin homeostasis. Since neither insulin resistance nor hyperglycemia was present in the ADKD mice (similar in this regard to the phenotype of mice hypomorphic for hepatic *Atgl*) [24], [48], [49], we investigated other mechanisms relating *Ildr2* to hepatic steatosis.

Localization of ILDR2 in the ER membrane, up-regulation of ER stress markers in the livers of ADOX mice and down-regulation in ADKD mice, along with the emerging relationship between hepatic lipid accumulation and ER stress in several metabolic disorders, including obesity, hepatic steatosis and type II diabetes [16], [31], [50], suggest that ILDR2 might have a role in cellular ER stress responses.

Three known pathways provide mechanisms whereby *Ildr2* regulation of hepatocyte lipid metabolism and ER stress could be achieved:

1. ILDR2 has a primary role in ER function, where ER stress produced by *Ildr2* knockdown leads to lipid accumulation. Overexpression of the ER stress chaperone BIP (GRP78) in *ob/ob* mice (as with *Ildr2*) reverses hepatic steatosis [51], and hypomorphic expression of UPR modulators *Atf6*, *Ire1α*, *Chop*, and *Crebh* in mouse models cause hepatic dyslipidemia [32], [33], [52], [53].
2. ILDR2 has a primary role in lipid metabolism, where *Ildr2* knockdown leads to lipid accumulation, which causes ER stress. Excess intracellular fatty acids induce ER stress in the liver via pathways affecting ER membrane integrity and calcium homeostasis [40], [54], increasing *Chop* expression [55], inducing PERK signaling [28] and stimulating CREBh-induced inflammation [56], [57]. Additionally, fatty acid–binding protein-4 (aP2) has a primary role in lipid metabolism and mitigates ER stress in macrophages [39].
3. ILDR2 is independently involved in both ER function and lipid metabolism, as has been suggested for the ER stress-related gene, *Xbp1*, a key transcription factor and effector of the UPR which is spliced by IRE1α in response to ER stress. *Xbp1* KO mice show reduced hepatic TG secretion and decreased fatty acid oxidation, along with down-regulation of key hepatic lipogenic genes [26]. While we have not shown that ILDR2 is a transcription factor, by acting on downstream signaling targets, including transcription factors, it could independently affect the UPR and lipid metabolism.

The broad down-regulation of lipid metabolism and ER stress genes in the ADKD mice at 10 days p.t. is consistent with studies of pharmacologically-induced ER stress, in which expression of genes involved in lipid metabolism and ER stress initially increases and then declines. This pattern has been observed both *in vitro* and *in vivo* for spliced *Xbp1*, *Chop*, *Bip*, lipogenic transcription factors, lipid droplet proteins, and TG synthesis genes [34]. If *Ildr2* knockdown induces ER stress in these mice, then by 10 days we may be observing the decline of previously up-regulated ER stress and metabolic genes.

Since it remains uncertain, which gene expression effects are primary in the pathogenesis of the hepatic steatosis, and which may be responses to the steatosis *per se*, the data obtained in the 3 and 10 day adenovirus transductions should be cautiously interpreted. Absence of a clear pattern in the differential responses of the canonical UPR pathways seen in the studies reported here suggests that these variable responses may reflect differences in the timing of the responses of specific molecules and pathways coupled with secondary effects of the accumulation of hepatic lipids [58].

*Ildr2*-mediated effects on lipid homeostasis and ER stress responses could account for both the hepatic steatosis observed in the ADKD animals reported here, and the reduced β-cell mass and accompanying glucose intolerance in the Chr 1 B6.DBA *ob/ob* congenic animals [1]. *Perk*–null mice develop ER stress specifically in the β-cell, with morphological abnormalities within the pancreatic ER leading to loss of β-cells, and hypoinsulinemic hyperglycemia [59]. Deregulation of lipid metabolism in a β-cell line impaired insulin secretion [60].

In a recent study, ILDR2, along with ILDR1 and ILDR3, was localized to tricellular junctions on the PM of mouse epithelial cells – specifically fibroblasts, mammary and retinal epithelia, and choroid plexus [61]. The authors propose that these molecules mediate macromolecular access through these “tight junctions”. However, no specific effort was made to visualize ILDR2 or other ILDR molecules in the ER. As has been described for the thyrotropin-releasing hormone receptor, which is localized to the PM in pituitary cells but to the ER and Golgi in non-pituitary cells [62], ILDR2 may localize primarily to the PM in epithelial cells, and to the ER in other cell types (e.g. hepatocytes, neurons, β-cells). Alternatively, subcellular distribution of members of this family of proteins may reflect cell type-specific splicing patterns, as reported for BAT3 [63].

## Materials and Methods

### Ethics Statement

All protocols were approved by the Columbia Institutional and Animal Care Use Committee (Permit Number: AAAC3125).

### Animal Care

Mice were housed in a vivarium maintained on a 12 hr –12 hr light-dark cycle, with *ad libitum* access to 5058 Purina PicoLab Mouse Diet 20 (9% fat) and water, unless otherwise stated. All mice were sacrificed at the same time-of-day (1000–1200 hr).

### Strains

Male 9-week-old C57BL/6J (B6) (Stock number 000664) and B6.V-Lep^ob^/J (ob/ob) (Stock number 000632) mice were obtained from Jackson Laboratories (Bar Harbor, ME) and allowed to adjust to conditions in our local colony for 1 week prior to starting experiments. Mice used to study the effects of feeding status and diet (Figure 8A) were fed high-fat chow (60% kcals from fat) at Jackson Laboratories from 6 weeks of age until purchase at 18 weeks of age. Mice were fed ad libitum high-fat chow (Research Diets D12492i) for 4 additional weeks.

### Metabolic Parameters

#### Body mass and composition

Weight was measured with a Vicon Vic-212 integrating laboratory scale (Acculab). Fat and lean mass were measured with a Minispec TD-NMR Analyzer (Bruker Optics), calibrated using mouse carcasses [64].

#### Serum

Blood was collected at sacrifice. Plasma was analyzed for glucose using an Autokit Glucose (Wako), for TG using an L-Type TG M Color A (Wako), for TCH using Cholesterol E (Wako), for FFA using HR Series NEFA-HR(2) Color Reagent B (Wako) and insulin using an Ultra Sensitive Mouse Insulin ELISA Kit (Crystal Chem). Glucose in living mice was measured with a FreeStyle Lite portable glucose meter (Abbott) using 3 µl blood from a capillary tail bleed. IPGTT was performed in the morning after overnight fast. Blood for fasting glucose analysis was collected by tail bleed. Mice were injected with 2 mg/g of glucose using a 50 mg/ml solution in autoclaved water. Blood was collected at 5, 15, 30, 60, and 120 minutes and glucose was measured with the Autokit Glucose.

### Plasma Lipid Profile and Triton Experiment

250 µl of pooled plasma from 6 mice fasted for 5 hr was used for FPLC analysis using 2 Sepharose 6 Fast Flow columns in series (Amersham Biosciences). The buffer contained 100 mM Tris and 0.04% NaN3, pH 7.5; a flow rate of 0.7 ml/min was used. TCH and TG levels of FPLC fractions were measured using Wako enzymatic kits. To block clearance of nascent lipoproteins, these mice were then injected with Triton WR1339 (0.5 mg/g body weight; Sigma-Aldrich) via tail vein. Blood samples were collected at 0, 30, 60, and 120 min post-injection. Initial plasma samples were used for TG quantification.

### Liver Glycogen

Liver fragments (0.1–0.2 g) were digested in 1 ml of 30% KOH at 95°C for 30 min; 0.2 ml of 2% Na_2_SO_4_ and 3.2 ml of 70% ethanol were added and the mixture was centrifuged for 30 min at 6800 RCF. Pellets (containing glycogen) were washed with 70% ethanol and resuspended in 0.5 ml of 0.2 M acetate buffer; 0.1 ml of the solution was incubated for 30 min at 55°C with 5 µl of amyloglucosidase (Sigma) and then incubated 5 min at 37°C with Autokit Glucose (Wako Diagnostics, Richmond, VA). Glycogen content was expressed as mg of glucose/g of wet liver.

### Liver Lipids

Whole lipids were extracted by Bligh-Dyer extraction [65]. In brief, 1.5 ml of chloroform:methanol (1∶2, v/v) with 0.4 ml of PBS was added to liver pieces (0.1–0.2 g) in a screw-capped glass test tube and mixed vigorously for 1 min. Vigorous mixing followed successive additions of 0.5 ml chloroform and 0.5 ml H_2_O. The mixture was centrifuged at 1800 RCF for 5 min and the lower (organic) phase, containing whole lipids, was collected and stored at −20°C until assay. TCH and TG were determined with a colorimetric kit (Wako; Cholesterol E 439-17501; L Type TG 461-08892 and 461-09092).

### HOMA-IR

HOMA2-IR (homeostasis model assessment-estimated insulin resistance) and HOMA-2-B% (homeostasis model assessment-β-cell function) were calculated using the HOMA calculator, http://www.dtu.ox.ac.uk/homacalculator/index.php, based on the nonlinear updated HOMA2 model [66], which takes account of variations in hepatic and peripheral glucose resistance, increases in the insulin secretion curve for plasma glucose concentrations >10 mmol/L (180 mg/dL) and the contribution of circulating proinsulin.

### Energy Expenditure

Energy expenditure was measured with a LabMaster-CaloSys-Calorimetry System (TSE Systems, Bad Homburg, Germany). O_2_ and CO_2_ measurements were taken every 26 min during a 72 hr period from 32, 10-week-old male mice (8 ADOX, 8 ADOX control, 8 ADKD, and 8 ADKD control). Mice were injected with the adenovirus on day 0 and placed in calorimeters from days 5–7. Because of stress related to transfer to the calorimetry chambers, only measurements taken within the last 48 hr were used to calculate total 24-hr energy expenditure (TEE; in kcal/24-hr) and respiratory exchange rate (RER = VCO_2_/VO_2_). Resting energy expenditure (REE in kcal/24-hr) was defined as the1-hr period of lowest energy expenditure. This coincided with the 1 hr of lowest total ambulatory activity (generally early afternoon), during the 48-hr period; this value was extrapolated to 24 hr. Non-resting energy expenditure (NREE) was calculated as the difference between TEE and REE (NREE = TEE – REE). Physical activity was measured by an infrared beam system integrated with the LabMaster system. Total activity (beam breaks) in X, Y, and Z axes was recorded every 26 min. The system is designed to differentiate between fine motor movement (defined as a single X or Y axis beam break), ambulatory movement (defined as the simultaneous breaking of two adjacent X or Y beams), and rearing, defined as the breaking of the Z axis infrared beam. Lights were off at night from 1900 to 0700 hr.

### Adenovirus Studies

#### Adenoviral expression vectors

Adenoviruses were prepared and amplified with the ViraPower Adenoviral Expression System (Invitrogen). Viral titers were determined by plaque-forming assays on HEK 293A cells. PCR-amplified, full-length Ildr2-cDNA was subcloned into the pENTR/D-TOPO vector using the pENTR Directional TOPO Cloning Kit (Invitrogen). After verifying the sequence, inserts were transferred into the pAd/CMV/V5-DEST vector by the Gateway system using LR Clonase II Enzyme Mix. Sequences corresponding to the shRNAs for Ildr2 and lacZ were cloned into pBlock-it (Invitrogen). The sequence of the shRNA for Ildr2 was: 5′-cac cGT TCA AAT CCT ACT GCC Aga cgt gtg ctg tcc gtC TGG CAG TAG GAT TTG AAC-3′, where the 5′ uppercase 18-nucleotide sequence corresponds to the coding strand in exon 2 for the amino acid sequence FKSYCQ.

#### Virus purification

To obtain virus particles, plasmids were linearized by Pac I digestion and transduced into HEK 293A cells with Lipofectamine 2000 using Opti-MEM medium. The transduced HEK 293A cells were incubated at 37°C in a 6 cm dish until the cells started to die (about 10 days). The cells and supernatant were harvested in a 50 ml tube and subjected to 3 freeze-thaw cycles to lyse the cells. The suspension was centrifuged at 1800 RCF for 15 min to eliminate cellular debris. The supernatant was collected and used to transduce a new 10 cm dish of HEK 293A cells. This process (grow, lyse, centrifuge, transduce a larger number of cells) was repeated until 20, 15-cm dishes, were incubated simultaneously. The cells and supernatant were collected and spun at low speed (200 RCF) for 2 min. The cells and 5 ml of supernatant were then subjected to 3 freeze-thaw cycles to lyse cells, followed by centrifugation at 1800 RCF for 15 min. A CsCl step gradient was set up with a lower layer of 4 ml of 1.4 g/ml CsCl and an upper layer of 3 ml of 1.2 g/ml CsCl. 5 ml of supernatant was over-layered and ultracentrifuged at 65,000 RCF for 90 min at 4°C. The 1.2 g/ml cesium chloride layer, containing virus, was extracted and dialyzed vs. 10 mM Tris/HCL at pH 8.0. Viral concentration was determined by OD260 assay.

#### Injection

Recombinant viruses were administered via tail vein injection and mice were sacrificed 3 days or 10 days p.t.

#### Real-Time qPCR

RNA was extracted with TRIzol acid-phenol reagent (Invitrogen) and purified with on-column DNase digestion using RNeasy Mini Kit (Qiagen). RNA integrity was verified by visual inspection of ethidium bromide stained electrophoresis gels and by OD260 nm*/*OD280 nm*>*1.9 and OD260 nm*/*OD230 nm*>*2.0. First strand cDNA synthesis was performed using 1 µg of total RNA each and the Sprint RT Complete-Random Hexamer kit (Clontech) according to the manufacturer’s instructions. Reverse transcription (RT) followed by PCR was used to analyze mRNA abundance in response to treatments. Primers for genes were designed to produce an amplification product which spanned at least one exon using the Universal Probe Library Assay Design Center (www.universalprobelibrary.com, Roche Applied Sciences); primers were synthesized by Invitrogen. qPCR analysis was performed on a LightCycler 480 (Roche) using the LightCycler 480 SYBR Green I Mastermix (Roche).

### Primers for PCR

Primers used for the PCR amplification of full-length *Ildr2*-cDNA

*Ildr2* Forward: caccATGGATAGGGTCGTGTTGGG

*Ildr2* Reverse: TCAGACTACAAGGGACATCCTGGTTGGAAAGTCACC

The first TCA in the reverse is the stop codon. The ATG in the forward is the start codon. Primers used in expression analysis are shown in **Table 10**.

**Table 10:** Primers used for expression analysis.

Gene	Forward	Reverse
*Ildr1*	TCATTGTCCTGCATTGGCTGA	CAACAGCGGGTAGGACAGCA
*Ildr2*	ACAGGGCTCGACGGTTAC	ACACCCACTCCAACACCAGC
*Ildr3*	TCACCATCACAGGAAATGCTGAC	GCTTCTGAGGTCCTGCCAAGG
*Pepck*	TGTCATCCGCAAGCTGAAGA	TTCGATCCTGGCCACATCTC
*Gck*	TCCCTGTAAGGCACGAAGACAT	ATTGCCACCACATCCATCTCA
*Glut2*	GGAACCTTGGCTTTCACTGTCTT	GGAACACCCAAAACATGTCGAT
*Gpat*	GGCTACGTCCGAGTGGATTTT	AACATCATTCGGTCTTGAAGGAA
*Mgat1*	CTGGTTCTGTTTCCCGTTGT	GGTGAATGTTCCTGGGTGAG
*Dgat1*	CCTCAGCCTTCTTCCATGAG	ACTGGGGCATCGTAGTTGAG
*Dgat2*	TCCAGCTGGTGAAGACACAC	GATGCCTCCAGACATCAGGT
*FAS*	ATCCTGGAACGAGAACACGATCT	AGAGACGTGTCACTCCTGGACTT
*ACC1*	GGGCACAGACCGTGGTAGTT	CAGGATCAGCTGGGATACTGAGT
*Pparg2*	TTCCACTATGGAGTTCATGCTTGT	TCCGGCAGTTAAGATCACACCTA
*Srebp1c*	CGGCGCGGAAGCTGT	TGCAATCCATGGCTCCGT
*Srebp2*	CTGCAGCCTCAAGTGCAAAG	CAGTGTGCCATTGGCTGTCT
*Ldlr*	TGGAGGATGAGAACCGGCT	GCACTGAAAATGGCTTCGTTTA
*Apob*	TCACCCCCGGGATCAAG	TCCAAGGACACAGAGGGCTTT
*Ppara*	CCTCAGGGTACCACTACGGAGT	GCCGAATAGTTCGCCGAA
*Cpt1a*	CCTGGGCATGATTGCAAAG	GGACGCCACTCACGATGTT
*Acox1*	CGATCCAGACTTCCAACATGAG	CCATGGTGGCACTCTTCTTAACA
*Mcad*	TGCTTTTGATAGAACCAGACCTACAGT	CTTGGTGCTCCACTAGCAGCTT
*Ucp2*	GACCTCATCAAAGATACTCTCCTGAA	ATCTCGTCTTGACCACATCAACAG
*Rxra*	GGCAAACATGGGGCTGAAC	GCTTGTCTGCTGCTTGACAGAT
*Fxra*	TGGGCTCCGAATCCTCTTAGA	TGGTCCTCAAATAAGATCCTTGG
*Fxrb*	GGGCTTAGAAAATCCAATTCAGATTA	CGTCCGGCACAAATCCTG
*Perk*	CCTTGGTTTCATCTAGCCTCA	ATCCAGGGAGGGGATGAT
*Atf6*	GGACGAGGTGGTGTCAGAG	GACAGCTCTTCGCTTTGGAC
*Ire1*	TGAAACACCCCTTCTTCTGG	CCTCCTTTTCTATTCGGTCACTT
*Atf4*	ATGATGGCTTGGCCAGTG	CCATTTTCTCCAACATCCAATC
*Xbp1*	TGACGAGGTTCCAGAGGTG	TGCAGAGGTGCACATAGTCTG
*Chop*	TCCCTGCCTTTCACCTTG	GCCCTGGCTCCTCTGTCA
*Bip*	CTGAGGCGTATTTGGGAAAG	TCATGACATTCAGTCCAGCAA

PCR primers used in experiments described in **Table 1**, **Figure 6**, and **Figure 7**.

### Construction of Tag Protein Fusions

N-terminal 3xFLAG *Ildr2* fusion construct. The *Ildr2* open reading frame from exon 2 was subcloned into p3xFLAG-CMV-8 (N-terminal FLAG with PPT LS, Sigma-Aldrich # E4151-20UG). *Ildr2* was amplified using a forward primer on the sequence coding for the first amino acids of exon 2 with a HindIII site (5′ ATT TAC AAG CTT CAG GTC ACA GTG CCT GAC AAG AAG AAG GT3′), and a reverse primer with an in-frame stop codon and EcoR1 restriction site at the end of *Ildr2* last exon (5′- CAT GCA GAA TTC TCA GAC TAC AAG GGA CAT CCT G -3′). The destination vector and the PCR amplified *Ildr2* sequence were digested with HindIII and EcoR1 (NEBiolabs) in NEBuffer EcoR1 and BSA at 37°C for 60 min, purified and ligated.

C-terminal -tagged ILDR2 mYFP construct. The *ildr2* open reading frame from exon 1 was subcloned into pmEYFP-N1 (Clontech # 6006-1). *Ildr2* was amplified using a forward primer on exon 1-including Kozak sequence- with a site for the restriction enzyme NheI (5′- ATC TTG CTA GCG GTA ATG GAT AGG GTC GTG TTG G-3′), and a reverse primer that bypass the stop codon and an EcoRI restriction site (5′- CAT GCA GAA TTC GGA CTA CAA GGG ACA TCC TG-3′). The destination vector and the PCR-amplified *Ildr2* sequence were digested with Nhe and EcoRI (NEBiolabs) in NEBuffer EcoRI+BSA at 37°C for 60 min, purified and ligated.

### Isolation of Hepatocytes

Hepatocytes were pooled from 5, 10-week-old C57BL/6J mice. The mice were anaesthetized with cocktail containing ketamine (100 mg/kg) and xylazine (20 mg/kg) and then laparotomized to expose the liver and the portal vein. A 25G winged needle attached to a 50 ml syringe filled with 37°C EGTA-Hanks solution (Hanks Balanced Salt Solution, Gibco; EGTA final concentration 0.5 mM) was inserted into the portal vein and clipped in place with a clamp at the root of the mesentery and the needle. After cutting the inferior vena cava, the liver was perfused with 30 ml EGTA-Hanks. Using a fresh syringe, the liver was then perfused with 20 ml of a solution containing collagenase (5 mM CaCl_2_ in Hanks with 1 mg/ml of collagenase type II Gibco # 17101-015) being careful not to inject bubbles. Liver was excised and placed in a 10 cm sterile dish on ice with 2–3 ml of collagenase solution and minced with scissors to remove visible blood clots. Minced livers were pooled and incubated for 5–10 min at 37°C and homogenized by pipetting up and down 20–30 times. Then 20 ml of RT Hanks balanced salt solution was added to the dish and mixed. The suspension was filtered through sterile gauze into a 50 ml conical tube, spun 1 min at 200 RCF at RT and aspirated to remove supernatant. Cells were resuspended in 20 ml Gibco HG medium (with 10% FBS, 1% Penicillin Streptomycin, 10 nm DEX, 100 nm insulin, and 0.1% Fungizone) and pipetted up and down 5 times. The suspension was filtered through a 100 um Nylon cell strainer (BD Falcon REF352360) and collected in 50 ml conical tube. The filtrate was centrifuged again at 200 RCF for 1 min at RT and aspirated to remove the supernatant. Cells were resuspended in 25 ml of Gibco HG medium and gently pipetted. Cells were counted with an Invitrogen Countess using trypan blue staining and then distributed at 3×10^6^ cells per 10 cm plate and incubated overnight at 37°C in a humidified 5% CO_2_ incubator before administering virus.

### Cell Line Studies

#### Cell microscopy, image acquisition

Cell cultures were prepared and maintained according to standard cell culture procedures. Hepa1c1c7 and GT1-7 cells were maintained in Dulbecco’s Modified Eagle Medium supplemented with 10% fetal calf serum using BD Falcon T75 cell culture flasks. For transient transfection, cells were harvested by Trypsin/EDTA digestion, seeded on coverslips (1×10^5^ per coverslip) and incubated for 24 h in a cell culture incubator at 37°C and 5% CO_2_. Transfection of plasmid DNA for GFP-tagged ILDR2 was performed with Lipofectamine 2000 according to the manufacturer’s instructions. In brief, cells were incubated with 500 ng plasmid DNA and 1.25 µl Lipofectamine 2000 in Opti-MEM (24-well plate format) over night before being analyzed. Transfection was up-scaled accordingly if other plate formats were used.

#### Confocal images

Transduced cells were washed with PBS, fixed in 4% paraformaldehyde at room temperature (rt) for 15 min, washed again with PBS, permeabilized with 0.1% Triton-X 100 (2 min at rt) if necessary and incubated with blocking buffer (5% normal goat serum and 0.05% Tween 20 in PBS) for 30 min. For immunostaining, cells were incubated overnight at 4°C with the corresponding specific antibody diluted in blocking buffer, washed with PBS, incubated for 1 hr at rt with 2 µg/ml Alexa Fluor 546 goat anti-mouse secondary antibody diluted in blocking buffer washed with PBS and mounted in ProLong Gold antifade reagent with DAPI as a nuclear staining marker. Samples were analyzed with a Confocal Laser Scanning Microscope (TCS SP2, Leica, Germany). When fluorescent proteins were used, GFP was excited at 488 nm and fluorescence was detected at 500–540 nm. YFP was excited at 514 nm and fluorescence was detected at 520–560 nm. DsRed was excited at 557 nm and fluorescence was detected at 592 nm. Alexa Fluor 546 was excited with the 543 nm line of the helium laser and fluorescence was detected at 555–700 nm. Cells were imaged with a 63.0x/1.25 HCX PL APO objective lens. Images were processed with Adobe Photoshop software. Images shown are stacks of several confocal sections.

#### siRNA reverse transfection

RNA interference-mediated gene knockdown was achieved using custom Stealth RNAi™ siRNA designed using the BLOCK-iT™ RNAi Designer software (Invitrogen), and its correspondent controls.10 nM RNAi duplex was diluted in Opti-MEM® I Reduced Serum Medium (Invitrogen) and 5 ul of Lipofectamine™ RNAiMAX (Invitrogen) was added to each well containing the diluted RNAi. After mixing, the complexes were incubated for 20 min at rt. After incubation, approximately 0.15–0.25×10^6^ cells per well in complete growth medium without antibiotics were added to the wells containing the siRNA-lipid complexes. Cells were then cultured in normal growth medium processed for RNA or protein extraction as indicated by experimental procedure.

#### Western blotting

Cells were lysed in M-PER Mammalian Protein Extraction reagent (Thermo Scientific) with protease (Calbiochem) and phosphatase inhibitor (Thermo Scientific). Equivalent amounts of protein (100 to 400 µg) from each sample were diluted in 1× LDS sample buffer (Invitrogen) containing 100 mM DTT and incubated for 10 min at 70°C. After denaturing, the mixture was cooled at rt for at least 15 min and the protein was resolved by SDS-PAGE. The gel was transferred onto polyvinylidene fluoride (PVDF) membrane and blocked by incubation for 1 hr at rt in a solution 5% bovine serum albumin fraction V (BSA) in Tris-buffered saline with Tween (TBST) at pH 7.4, followed by overnight incubation at 4°C with primary antibody in 5% BSA/TBST. Following 3 15-min washes with TBST, membranes were incubated for 1 hr at rt with the corresponding secondary antibody and washed 3 more times in TBST. Membranes were developed using a chemiluminescence assay system and proteins were visualized using Kodak exposure film. Membranes were stripped using Restore™ PLUS Western blot stripping buffer (Thermo Scientific) with vigorous agitation for 10 min at rt, followed by 3 TBST washes.

### Statistical Analysis

Two tailed T tests were performed using Microsoft Excel.
